# Representation-aware conformal prediction for reliable uncertainty quantification in drug-target affinity

**DOI:** 10.1093/bioinformatics/btag771

**Published:** 2026-10-13

**Authors:** Xinya Li, Yaxin Zhang, Ximin Zeng, Danping Zhou, Yue Cheng, Shaoping Shi

**Affiliations:** Department of Mathematics, School of Mathematics and Computer Sciences, Nanchang University, Nanchang 330031, China; Department of Mathematics, School of Mathematics and Computer Sciences, Nanchang University, Nanchang 330031, China; Department of Mathematics, School of Mathematics and Computer Sciences, Nanchang University, Nanchang 330031, China; Department of Mathematics, School of Mathematics and Computer Sciences, Nanchang University, Nanchang 330031, China; Department of Mathematics, School of Mathematics and Computer Sciences, Nanchang University, Nanchang 330031, China; Department of Mathematics, School of Mathematics and Computer Sciences, Nanchang University, Nanchang 330031, China

## Abstract

**Motivation:**

Quantifying predictive uncertainty is essential for computational drug discovery to reduce experimental costs and false positives. However, most drug-target affinity (DTA) models focus on improving prediction accuracy without reliable uncertainty estimates.

**Results:**

We propose representation-aware conformal prediction (RCP)-DTA, a representation-aware conformal prediction framework that constructs adaptive prediction intervals based on feature similarity. By constructing local calibration sets, our approach generates sample-specific prediction intervals that achieve empirical coverage close to the nominal confidence level. To improve representation quality, RCP-DTA employs a cross-modal architecture that integrates pretrained sequence representations and structural information. Binding site context is incorporated via virtual pocket feedback to enhance residue representations, followed by gated fusion for adaptive integration of sequence and structural modalities. Evaluations across multiple DTA benchmark datasets demonstrate that RCP-DTA achieves competitive affinity prediction performance while providing reliable uncertainty quantification.

**Availability and implementation:**

Code and data are available at: https://github.com/Lee515628/RCP-DTA

## 1 Introduction

Identifying lead compounds with both high affinity and specificity across the vast chemical space remains a central hurdle in drug discovery (Gorgulla *et al.* 2020). Drug-target affinity (DTA) offers a quantitative measure of binding strength that has become essential to accelerating drug design workflows (Debnath *et al.* 2025). Experimental assays, while reliable, are difficult to scale to large libraries because of their cost and limited throughput (Poli *et al.* 2020). Molecular docking can simulate physical interactions with reasonable fidelity, yet its computational burden is heavy and its accuracy depends critically on the quality of available structures (Jakhar *et al.* 2020). Deep learning has gained traction as a way around these bottlenecks, enabling end-to-end DTA prediction that is both rapid and scalable when trained on large molecular datasets (Ren *et al.* 2023).

The evolution of representation learning has reshaped DTA prediction over the past several years. DeepDTA (Öztürk *et al.* 2018) was among the first to apply 1D convolutional neural networks directly to drug and protein sequences, establishing a baseline that many later methods built upon. MT-DTA (Zhu *et al*. 2023a) enhanced sequence representations by introducing mutual transformer and associative learning mechanisms to capture complex dependencies between drug and target representations. GraphDTA (Nguyen *et al.* 2021) further incorporated molecular graph representations to model bond-level topological information beyond sequence features. Subsequent studies explored more advanced graph representation strategies. TDGraphDTA (Zhu *et al*. 2023b) investigated multiscale information interaction and graph optimization, while MDCT-DTA (Zhu *et al.* 2024) introduced multiscale diffusion and interactive learning mechanisms. RRGDTA (Zhu *et al.* 2025b) further employed rotary encoding and information retention mechanisms to preserve informative molecular features and capture long-range dependencies. With the increasing availability of protein structures, 3D architectures have become an important direction in DTA prediction. 3DProtDTA (Voitsitskyi *et al.* 2023) introduced spatial coordinates into molecular representations. Geo-PLA (Chen *et al.* 2025) further exploited geometric equivariant graph learning with local-global structure awareness to capture rotation-invariant protein–ligand complex representations. Beyond affinity prediction, DiffFBDD (Zheng *et al.* 2025) incorporated equivariant 3D representations and conditional diffusion models for pocket-guided drug generation. Meanwhile, within DTA prediction, increasing attention has been directed toward biologically relevant local regions and multimodal integration. PocketDTA (Zhao *et al.* 2024) focused on local binding pocket geometry to reduce noise from irrelevant structural regions, while MMPD-DTA (Wang *et al.* 2025) introduced a pocket-drug graph to explicitly model local target–drug interactions. DeepTGIN (Wang *et al.* 2024) combined transformer encoders and graph isomorphism networks to capture complementary sequence and structural features. TriM-DTA (Zhu *et al*. 2025a) further integrated sequence, topological, and geometric information through hierarchical multimodal fusion. MultiMolCGC (Xu *et al.* 2025) introduced a multimodal multitask graph attention framework with customized gating mechanisms to integrate diverse molecular representations for accurate affinity prediction. Most of these methods optimize point-prediction accuracy and do not report calibrated uncertainty, particularly under distribution shift (Tarasov *et al.* 2024). For out-of-distribution (OOD) samples, confidence cannot be inferred directly from the magnitude of a point prediction.

Uncertainty quantification (Abdar *et al.* 2021) is essential for trustworthy DTA prediction, as it improves reliability in practical screening. Recent advancements have significantly propelled this field forward. KDBNet (Luo *et al.* 2023) integrates 3D structural learning with uncertainty recalibration to facilitate data-efficient active learning. EviDTI (Zhao *et al.* 2025) leverages evidential deep learning to directly quantify prediction uncertainty without relying on random sampling, enabling uncertainty-aware ranking of candidate interactions. Yet, many such existing approaches still rest on specific model assumptions or architectural modifications. Conformal prediction (CP) offers a way around this, providing distribution-free prediction intervals with guaranteed coverage (Vovk *et al.* 2005). Because these intervals contain the true label at a predefined confidence level, CP is particularly attractive for high-stakes settings like drug discovery (Alvarsson *et al.* 2021). Recent work has explored combining graph neural networks with quantile regression to tighten uncertainty estimates (Li *et al.* 2024), while others have introduced dynamic applicability domains that adaptively assess prediction coverage (Oršolić and Šmuc 2023). A common thread in localized calibration and applicability-domain methods is their reliance on heuristic similarity measures, such as Tanimoto coefficients or sequence similarity. These proxies often fall short in capturing the complexity of structure–activity relationships. Compounding this issue, prediction errors are frequently heteroscedastic across the feature space (Sousa *et al.* 2024), which makes reliable uncertainty estimation even harder. Global residual quantiles cannot adapt interval width to local variation, whereas predefined similarities may not align with prediction error. These limitations motivate calibration that adapts to the local structure of learned representations.

To address these limitations, we propose representation-aware conformal prediction (RCP)-DTA, a representation-aware CP framework that reformulates uncertainty quantification as a localized calibration problem in the learned feature space. Rather than applying a single global calibration rule, RCP-DTA performs neighborhood-conditioned calibration so that prediction intervals can adapt to local data complexity and heterogeneous error patterns. The framework uses representation-aware local residual quantiles to adapt interval width; coverage and interval efficiency are evaluated empirically.

Localized calibration requires representations in which nearby drug-target pairs have similar error behavior. RCP-DTA adopts a dual-stream strategy to integrate global semantic information with local structural context. Pretrained models supply sequence-based representations of drugs and proteins. To better characterize the binding interface, we introduce a virtual pocket feedback mechanism that encodes the physicochemical properties of binding pockets and propagates this information to the constituent residues. Structural features are then refined by attending to sequence representations through cross-modal attention, allowing functional and spatial information to be fused within a single representation. A gated fusion module combines the two modalities via element-wise gating, giving the model the flexibility to balance global functional semantics against local structural context depending on the input. These biologically informed representations provide the foundation for constructing local neighborhoods that are meaningful for calibration, which in turn is essential for reliable uncertainty estimation in DTA prediction. Experiments on multiple DTA benchmark datasets demonstrate that RCP-DTA achieves competitive affinity prediction performance. Moreover, evaluations on the Davis and KIBA datasets show that RCP-DTA provides improved uncertainty calibration and interval efficiency compared with baseline CP methods. We further benchmarked the framework across several backbone architectures and found that RCP consistently produces narrow, informative prediction intervals while maintaining empirical coverage. Finally, we also validated the method on epidermal growth factor receptor (EGFR) and human epidermal growth factor receptor 2 (HER2) case studies, where RCP-DTA produced well-calibrated intervals consistent with known binding behaviors.

## 2 Materials and methods

### 2.1 Datasets and preparation

We evaluated RCP-DTA on widely used benchmarks, including Davis (Davis *et al.* 2011), KIBA (Tang *et al.* 2014), Metz (Metz *et al.* 2011), and BindingDB (Gilson *et al.* 2016). Davis and KIBA are the primary benchmarks for affinity prediction and uncertainty evaluation. Metz and BindingDB provide additional regression evaluations. Detailed descriptions of dataset preprocessing are provided in Material 1.1 and Table 1, available as supplementary data at *Bioinformatics* online, while additional results on Metz and BindingDB are reported in Table 2, available as supplementary data at *Bioinformatics* online.

**Table 1:** Performance comparison of RCP-DTA and SOTA models on Davis and KIBA datasets.^a^

Dataset	Model	MSE↓	CI↑	${r_{m}}^{2}$ **↑**	Pearson↑	Spearman↑
Davis	DeepDTA	0.264 ± 0.013	0.877 ± 0.004	0.648 ± 0.024	0.819 ± 0.011	0.675 ± 0.001
	GraphDTA	0.272 ± 0.013	0.869 ± 0.003	0.637 ± 0.034	0.821 ± 0.010	0.666 ± 0.007
	AttentionDTA	0.223 ± 0.018	0.892 ± 0.003	0.670 ± 0.031	0.848 ± 0.010	0.697 ± 0.002
	3DProtDTA	0.192 ± 0.007	0.900 ± 0.005	0.701 ± 0.019	0.874 ± 0.005	0.705 ± 0.013
	PocketDTA	0.194 ± 0.009	0.896 ± 0.006	0.700 ± 0.019	0.873 ± 0.003	0.699 ± 0.014
	ColdstartCPI(Reg)	0.224 ± 0.011	0.881 ± 0.005	0.691 ± 0.012	0.853 ± 0.017	0.678 ± 0.006
	PMMR	0.233 ± 0.017	0.887 ± 0.009	0.632 ± 0.045	0.849 ± 0.008	0.687 ± 0.020
	MambaTransDTA	0.226 ± 0.015	0.883 ± 0.006	0.656 ± 0.021	0.847 ± 0.010	0.681 ± 0.010
	TSEDTA	0.213 ± 0.011	0.883 ± 0.002	0.676 ± 0.019	0.856 ± 0.012	0.686 ± 0.009
	RCP-DTA	**0.190 **±** 0.016**	**0.902 **±** 0.003**	**0.728 **±** 0.033**	**0.875 **±** 0.007**	**0.709 **±** 0.011**
KIBA	DeepDTA	0.240 ± 0.064	0.826 ± 0.032	0.635 ± 0.096	0.804 ± 0.063	0.790 ± 0.055
	GraphDTA	0.251 ± 0.018	0.815 ± 0.010	0.624 ± 0.040	0.803 ± 0.015	0.773 ± 0.020
	AttentionDTA	0.171 ± 0.004	0.863 ± 0.003	0.731 ± 0.008	0.867 ± 0.004	0.851 ± 0.005
	3DProtDTA	0.157 ± 0.005	0.871 ± 0.001	0.765 ± 0.002	0.880 ± 0.001	0.862 ± 0.004
	PocketDTA	0.157 ± 0.004	0.879 ± 0.002	0.713 ± 0.008	0.881 ± 0.001	0.870 ± 0.003
	ColdstartCPI(reg)	0.170 ± 0.004	0.874 ± 0.001	0.700 ± 0.016	0.870 ± 0.007	0.857 ± 0.006
	PMMR	0.227 ± 0.024	0.837 ± 0.016	0.574 ± 0.059	0.828 ± 0.016	0.805 ± 0.027
	MambaTransDTA	0.202 ± 0.011	0.863 ± 0.008	0.623 ± 0.026	0.848 ± 0.009	0.841 ± 0.010
	TSEDTA	0.171 ± 0.008	0.874 ± 0.013	0.730 ± 0.021	0.875 ± 0.006	0.860 ± 0.009
	RCP-DTA	**0.139 **±** 0.006**	**0.887 **±** 0.002**	**0.770 **±** 0.008**	**0.893 **±** 0.004**	**0.882 **±** 0.003**

a Bold values indicate the best performance.

**Table 2:** Generalization performance of RCP-DTA and baseline models under Cold-Start Scenarios on the KIBA dataset.^a^

Setting	Methods	MSE↓	CI↑	${r_{m}}^{2}$ **↑**	Pearson↑	Spearman↑
Unseen drugs	DeepDTA	0.438 ± 0.037	0.733 ± 0.020	0.360 ± 0.067	0.625 ± 0.045	0.602 ± 0.038
	GraphDTA(GIN)	0.410 ± 0.020	0.744 ± 0.013	0.393 ± 0.044	0.650 ± 0.022	0.625 ± 0.032
	3DProtDTA	0.369 ± 0.036	0.765 ± 0.009	0.444 ± 0.043	0.700 ± 0.028	0.663 ± 0.021
	ColdstartCPI(Reg)	0.374 ± 0.015	0.762 ± 0.001	**0.445 ± 0.010**	0.696 ± 0.019	0.661 ± 0.024
	MambaTransDTA	0.472 ± 0.037	0.735 ± 0.013	0.314 ± 0.035	0.614 ± 0.029	0.602 ± 0.026
	RCP-DTA	**0.365 **±** 0.020**	**0.772 **±** 0.010**	0.438 ± 0.045	**0.707 **±** 0.020**	**0.681 ± 0.019**
Unseen targets	DeepDTA	0.425 ± 0.084	0.701 ± 0.026	0.377 ± 0.040	0.624 ± 0.038	0.518 ± 0.058
	GraphDTA(GIN)	0.529 ± 0.105	0.647 ± 0.047	0.228 ± 0.078	0.488 ± 0.080	0.390 ± 0.118
	3DProtDTA	**0.370 **±** 0.026**	0.714 ± 0.025	0.422 ± 0.045	0.664 ± 0.029	0.575 ± 0.034
	ColdstartCPI(Reg)	0.402 ± 0.031	0.723 ± 0.018	0.412 ± 0.041	**0.702 ± 0.012**	0.568 ± 0.049
	MambaTransDTA	0.487 ± 0.066	0.694 ± 0.012	0.284 ± 0.011	0.583 ± 0.010	0.503 ± 0.033
	RCP-DTA	0.375 ± 0.035	**0.734 **±** 0.007**	**0.425 **±** 0.019**	0.682 ± 0.035	**0.585 **±** 0.023**
Dual cold-start	DeepDTA	0.621 ± 0.107	0.603 ± 0.030	0.130 ± 0.062	0.365 ± 0.094	0.279 ± 0.079
	GraphDTA(GIN)	0.717 ± 0.150	0.578 ± 0.020	0.070 ± 0.041	0.264 ± 0.091	0.214 ± 0.079
	3DProtDTA	0.587 ± 0.040	0.626 ± 0.048	0.225 ± 0.030	0.432 ± 0.047	0.406 ± 0.046
	ColdstartCPI(Reg)	0.582 ± 0.055	0.633 ± 0.015	0.177 ± 0.068	0.457 ± 0.064	0.357 ± 0.043
	MambaTransDTA	0.661 ± 0.127	0.608 ± 0.018	0.116 ± 0.056	0.352 ± 0.099	0.293 ± 0.005
	RCP-DTA	**0.542 **± 0.088	**0.648 **± 0.025	**0.235 **± 0.042	**0.462 **± 0.066	**0.416 **± 0.020

a Bold values indicate the best performance.

Protein structures were retrieved from the AlphaFold2 database (Varadi *et al.* 2024). For proteins without available structures, predictions were generated using ColabFold (Mirdita *et al.* 2022). Binding pockets were identified using P2Rank (Krivák and Hoksza 2018) to provide localized structural context for downstream modeling. Rather than restricting the model to pocket regions alone, the full protein structure is retained, while pocket-level information is incorporated to enhance residue representations. A detailed analysis of structural confidence across protein regions is provided in Fig. 1, available as supplementary data at *Bioinformatics* online.

**Figure 1:**
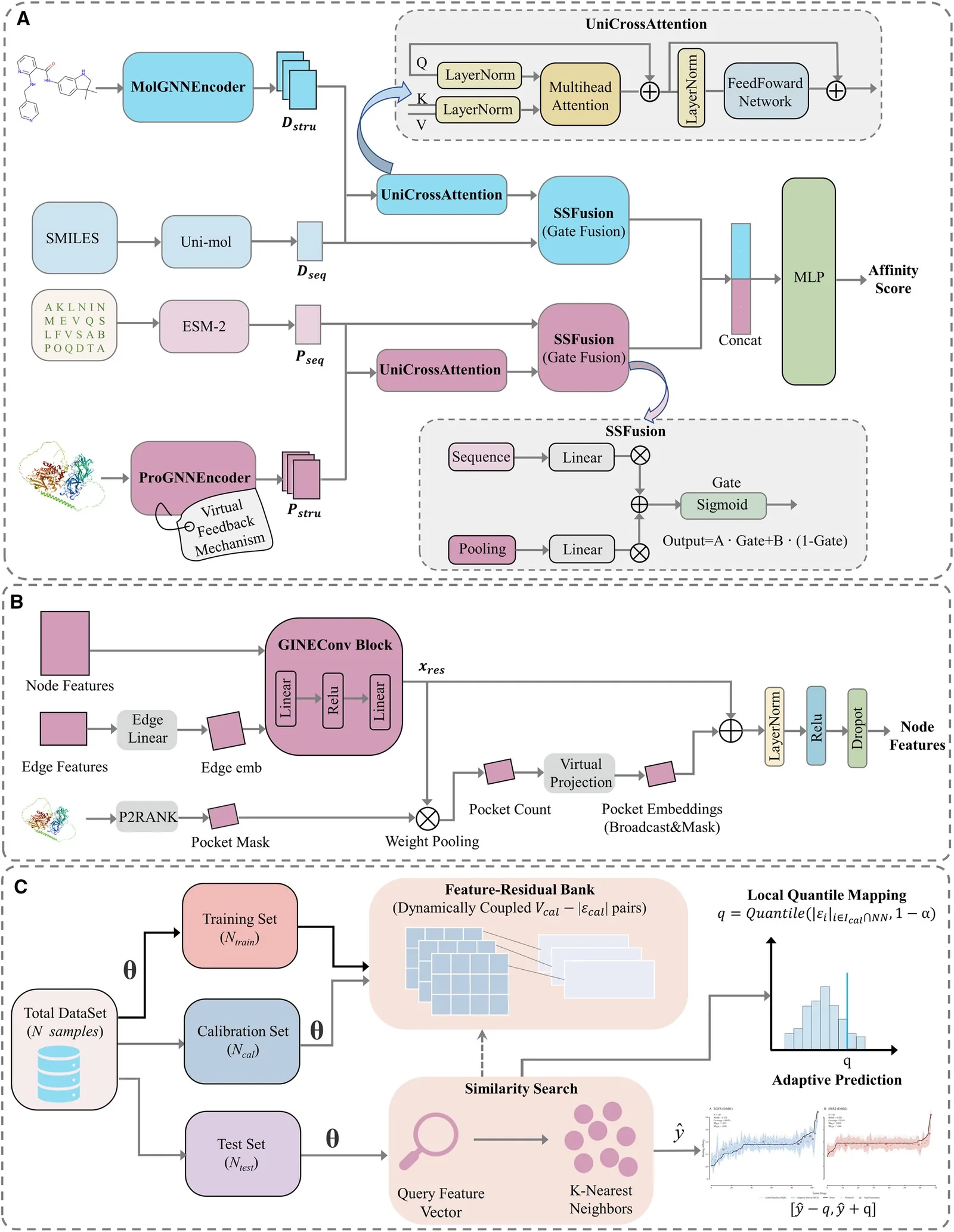
Overview of the RCP-DTA framework. (A) Multimodal fusion architecture for DTA prediction. Sequence and structural features are extracted and integrated via cross-modal attention and gated fusion. (B) Protein structure encoder with virtual pocket feedback, which propagates pocket-level information to residue features for modeling local binding interfaces. (C) Representation-aware CP for adaptive uncertainty estimation, based on similarity-driven local calibration in the learned feature space.

### 2.2 Model architecture

The overall architecture of RCP-DTA is shown in Fig. 1. The model adopts a multimodal dual-stream design to learn representations of drug-target pairs. In the feature encoding stage (Fig. 1A and B), sequence and structural features are extracted separately and subsequently integrated via cross-modal attention and gated fusion. The resulting drug and protein representations are concatenated for affinity prediction. A representation-aware CP module (Fig. 1C) is then applied to provide both point estimates and calibrated uncertainty intervals.

#### 2.2.1 Drug feature encoding

Drug representations are learned through a dual-stream encoding scheme that captures both global semantic information and local structural characteristics.

##### 2.2.1.1 SMILES-based representation

We employ the pretrained Uni-Mol model (Zhou *et al.* 2023) to obtain a global representation from the SMILES input $D_{\mathrm{smi}}$. The encoder outputs a latent embedding, where the [CLS] token is used as the molecular-level representation:


(1) Hdseq=UniMolEncoder(Dsmi)∈R512

This representation is projected into a unified feature space via a two-layer feedforward network:


(2) Dseq=Wd2·ReLU(Dropout(ReLU(Wd1Hdseq+bd1)))+bd2

where $W_{d1}\in R^{({2d}_{\mathrm{drug}})\times 512}$, $W_{d2}\in R^{d_{\mathrm{drug}}\times ({2d}_{\mathrm{drug}})}$, and $d_{\mathrm{drug}}$ = 256.

##### 2.2.1.2 Structure-based representation

To capture local chemical environments, each drug is represented as a molecular graph $G_{D}=(V_{D},E_{D})$ constructed using RDKit (Landrum 2015). Node features $x_{i}^{d}$ and edge features $e_{ij}^{d}$ encode physicochemical properties (Table 3, available as supplementary data at *Bioinformatics* online).

**Table 3:** Comparison of global and localized CP methods on Davis and KIBA datasets.^a^

α	Method	Davis			KIBA		
		Coverage↑	Width↓	Score↓	Coverage↑	Width↓	Score↓
0.05	CP	0.952 ± 0.004	1.265 ± 0.031	2.092 ± 0.058	0.950 ± 0.002	0.967 ± 0.013	1.615 ± 0.033
	CQR	0.951 ± 0.005	**1.187 ± 0.030**	2.025 ± 0.051	0.949 ± 0.002	0.947 ± 0.008	1.597 ± 0.036
	dAD(CV)	0.992 ± 0.003	4.296 ± 0.260	4.502 ± 0.273	**0.970 ± 0.001**	4.218 ± 0.057	4.895 ± 0.074
	dAD(NN)	**0.995 ± 0.001**	4.613 ± 0.050	4.729 ± 0.054	**0.970 ± 0.001**	4.202 ± 0.047	4.867 ± 0.063
	RCP_0.05	0.961 ± 0.004	1.267 ± 0.031	**1.926 ± 0.051**	0.949 ± 0.002	0.891 ± 0.011	**1.527 ± 0.036**
	RCP_0.10	0.956 ± 0.003	1.201 ± 0.032	1.932 ± 0.052	0.948 ± 0.002	**0.887 ± 0.011**	1.538 ± 0.035
0.1	CP	0.906 ± 0.005	0.784 ± 0.013	1.518 ± 0.038	0.897 ± 0.002	0.638 ± 0.003	1.196 ± 0.017
	CQR	0.906 ± 0.008	0.748 ± 0.030	1.468 ± 0.036	0.899 ± 0.003	0.640 ± 0.007	1.184 ± 0.019
	dAD(CV)	0.986 ± 0.002	2.950 ± 0.054	3.075 ± 0.038	0.935 ± 0.003	3.108 ± 0.037	3.951 ± 0.031
	dAD(NN)	**0.989 ± 0.001**	3.131 ± 0.071	3.233 ± 0.075	**0.936 ± 0.003**	3.139 ± 0.037	3.395 ± 0.030
	RCP_0.05	0.921 ± 0.003	0.733 ± 0.017	**1.356 ± 0.035**	0.899 ± 0.003	0.592 ± 0.005	**1.134 ± 0.019**
	RCP_0.10	0.919 ± 0.004	**0.723 ± 0.016**	1.377 ± 0.036	0.895 ± 0.003	**0.586 ± 0.005**	1.143 ± 0.018
0.2	CP	0.806 ± 0.007	0.373 ± 0.010	1.023 ± 0.023	0.798 ± 0.005	0.384 ± 0.004	0.847 ± 0.009
	CQR	0.806 ± 0.011	0.401 ± 0.010	0.999 ± 0.024	0.798 ± 0.003	0.391 ± 0.003	0.843 ± 0.009
	dAD(CV)	0.970 ± 0.003	1.146 ± 0.050	1.602 ± 0.052	0.864 ± 0.003	2.014 ± 0.017	3.012 ± 0.007
	dAD(NN)	**0.971 **±** 0.002**	1.491 ± 0.034	1.630 ± 0.044	**0.865 ± 0.003**	2.063 ± 0.016	3.038 ± 0.007
	RCP_0.05	0.832 ± 0.010	0.362 ± 0.007	**0.893 ± 0.024**	0.794 ± 0.003	0.357 ± 0.003	**0.807 ± 0.009**
	RCP_0.10	0.830 ± 0.009	**0.349 ± 0.008**	0.908 ± 0.025	0.790 ± 0.004	**0.352 ± 0.003**	0.814 ± 0.008

a Bold values indicate the best performance. RCP_ρ denotes the proposed method with neighborhood proportion *ρ*, which determines the number of nearest neighbors used for local quantile estimation as a fraction of the calibration set.

A MolGNNEncoder based on Graph Isomorphism Networks with edge features (GINE; Xu *et al.* 2019) is applied to refine these representations:


(3) xi(l)=MLP(l)((1+ϵ(l))·xi(l−1)+∑j∈N(i)ReLU(xj(l−1)+eijd,(l))),

where $x_{i}^{\left(l\right)}\in R^{d_{\mathrm{drug}}}$ denotes the node representation at layer l, j∈N(i) contains its direct neighbors, and $e_{ij}^{d,(l)}$ is the transformed edge feature. With a learnable $\epsilon^{\left(l\right)}$ and ${\mathrm{MLP}}^{\left(l\right)}$, this yields the final molecular-level node representation $D_{\mathrm{stru}}\in R^{d_{\mathrm{drug}}\times L_{d}}$ where $L_{d}$ indicates the number of atoms.

#### 2.2.2 Protein feature encoding

For proteins, a parallel dual-stream scheme is employed to encode both functional sequence signals and spatial structural context.

##### 2.2.2.1 Sequence-based representation

We employ the pretrained ESM-2 model (Lin *et al.* 2023) to encode the input protein sequence S. The encoder produces residue-level embeddings:


(4) Hres=ESMEncoder(S)∈RL×1280

where L is the actual sequence length (with sequences truncated or padded to $L_{\max}$). A fixed-size representation is obtained by averaging over residue embeddings:


(5) Hpseq=1L∑i=1LHires

This representation is projected into a lower-dimensional space via a two-layer feedforward network:


(6) Pseq=Wp2·ReLU(Dropout(ReLU(Wp1Hpseq+bp1)))+bp2

where $W_{p1}\in R^{({2d}_{\mathrm{pro}})\times 1280}$, $W_{p2}\in R^{d_{\mathrm{pro}}\times ({2d}_{\mathrm{pro}})}$, and $d_{\mathrm{pro}}$ = 256 denote the embedding dimension.

##### 2.2.2.2 Structure-based representation

To model spatial interactions, we construct a residue-level graph $G_{P}=(V_{P},E_{P})$, where nodes correspond to residues and edges connect residue pairs whose $C_{\alpha }$ atoms are within 8 Å. Node features encode amino acid identity, positional encoding, position-specific scoring matrix (Jeong *et al.* 2011), and backbone dihedral angles, as detailed in Table 4, available as supplementary data at *Bioinformatics* online. Pairwise distances are expanded into continuous edge features using radial basis functions.

A ProGNNEncoder (Fig. 1B) is employed to refine residue representations using GINE layers. At layer l, node features are updated as follows:


(7) hi(l)=MLP(l)((1+ϵ(l))·hi(l−1)+∑j∈N(i)ReLU(hj(l−1)+eijp,(l)))

where $h_{i}^{\left(l\right)}\in R^{d_{\mathrm{pro}}}$ is the node feature and $e_{ij}^{p,(l)}$ aligns the edge representation.

To incorporate binding site context, a virtual pocket feedback mechanism is introduced. Binding pockets are predicted using P2Rank (Krivák and Hoksza 2018), and the top-3 pockets ranked by probability are retained. A residue pocket assignment matrix $M=[M_{it}]\in {\left\{0,1\right\}}^{N_{p}\times T}$ is then defined, where $M_{it}=1$ indicates that residue i belongs to pocket t. Pocket level embeddings are then computed by aggregating residue features:


(8) Pt(l)=∑i=1Np(Mit·hi(l−1))∑i=1NpMit+γ, t∈{1,…,T}

These pocket representations are then propagated back to residue nodes:


(9) Δhi(l)=∑t=1TMit·(Wp(l)Pt(l))

where $W_{p}^{\left(l\right)}$ is a learnable projection matrix that maps pocket embeddings to node feature space. The updated node features are obtained via residual fusion and normalization:


(10) h∼i(l)=ReLU(Dropout(LN(hi(l)+Δhi(l))))

Stacking three GINE layers yields the final structural representation $P_{\mathrm{stru}}\in R^{{N_{p}\times d}_{\mathrm{pro}}}$, where $N_{p}$ denotes the number of protein residues.

#### 2.2.3 Cross-modal alignment and gated fusion

To integrate structural representations with pretrained semantic features, we employ an asymmetric cross-modal attention (Vaswani *et al.* 2017) mechanism (UniCrossAttention), where structural features attend to sequence representations. After layer normalization, structural and sequence features are projected into query, key, and value spaces:


(11) (Q,K,V)=(LN(Xstru)WQ,LN(Xseq)Wk,LN(Xseq)Wv)

where $Q\in R^{L_{\mathrm{stru}}\times d_{k}}$, and $K,V\in R^{L_{\mathrm{seq}}\times d_{k}}$. The attention operation is defined as follows:


(12) Att(Q,K,V)=Softmax(QKTdk)V

The output is refined through residual connections (He *et al.* 2016) and a feed-forward network:


(13) Xattn=Xstru+Dropout(Att(Q,K,V))

(14) Xupdated=Xattn+FFN(LN(Xattn))

To integrate sequence and structural information, a gated fusion module (SSFusion) is applied. The updated node features are first aggregated into a graph-level representation via average pooling:


(15) $$\begin{array}{c} Z_{pool}= \end{array}LN(\frac{1}{N}\sum_{i=1}^{N}X_{updated}^{\left(i\right)})$$

A dynamic gating factor g is learned to regulate the information flow between the sequence semantic stream $X_{\mathrm{seq}}$ and the structural topology stream $Z_{\mathrm{pool}}$:


(16) g=σ(W1Xseq+W2Zpool+b)

where σ(·) denotes the Sigmoid function. The module then performs an element-wise weighted fusion to obtain the final representation $Z_{\mathrm{final}}$:


(17) Zfinal=g⊙Xseq+(1-g)⊙Zpool,

where ⊙ represents the Hadamard product. This gating mechanism automatically balances the contributions of deep semantic embeddings and specific geometric matching constraints across different proteins, thereby improving model generalization.

### 2.3 Representation-aware CP

Following multimodal feature integration, the model concatenates the final drug and protein representations to form a joint embedding of the drug-target complex:


(18) Zcomplex=[Dfinal||Pfinal],

Layer normalization standardizes the concatenated vector to produce $Z_{\mathrm{norm}}$, which is then processed by a three-layer fully connected network with hidden dimensions 512 and 256. We employ ELU (Clevert *et al.* 2016) to address the numerical instability from wide-range affinity values:


(19) y^=Wf3·ELU(Wf2·ELU(Wf1·Znorm+bf1)+bf2)+bf3

where $W_{f1}\in R^{512\times {(d}_{\mathrm{drug}}+d_{\mathrm{pro}})}$, $W_{f2}\in R^{256\times 512}$, and $W_{f3}\in R^{1\times 256}$ are learnable weights with corresponding biases.

To quantify predictive uncertainty, RCP-DTA adopts a representation-aware CP framework. The working assumption is that nearby samples in the learned representation space have locally similar residual distributions; this assumption is assessed empirically rather than guaranteed. As illustrated in Fig. 1C, RCP constructs a non-parametric residual bank to achieve sample-specific interval estimation. We partition the dataset into training, validation, calibration, and test sets (7:1:1:1). The calibration set, which is not used during model training, is employed to construct a feature-residual bank: ${\left\{(Z_{j},\varepsilon_{j})\right\}}_{j=1}^{N_{\mathrm{cal}}}$, where $\varepsilon_{i}=|y_{i}-{\hat{y}}_{i}|$ denotes the nonconformity score.

To enable calibration that is specific to each sample, we perform nearest neighbor retrieval in the learned representation space. All calibration embeddings are L2-normalized and indexed using the Faiss library (Douze *et al.* 2026) for efficient similarity search. The size of the local calibration set is controlled by a neighborhood ratio $\left.\rho \in (0,1\right]$, which determines the number of nearest neighbors as a proportion of the calibration set:


(20) K=max⁡1,Ncal·ρ

For a test sample $x_{\mathrm{test}}$, its representation $z_{\mathrm{test}}$ is extracted and normalized to ${\tilde{z}}_{\mathrm{test}}$. A local calibration set is then constructed by retrieving its K nearest neighbors (Cunningham and Delany 2022) from the normalized calibration set:


(21) NK(xtest)=KNN(z∼test,{Z∼i}i=1Ncal)

Traditional CP relies on a global quantile, which ignores the local heterogeneity of data distributions. In contrast, RCP achieves fine-grained calibration by constructing a local calibration set for each test point. Given a significance level α, the model computes the 1-α empirical quantile of the local residuals:


(22) q^test=Quantile({εi}i∈NK(xtest), 1-α)

The final prediction interval is then defined as follows:


(23) C^α(xtest)=[y^test-q^test,y^test+q^test].

RCP-DTA is designed as a local calibration strategy, and its uncertainty estimates are evaluated by empirical coverage rather than strict distribution-free conformal guarantees.

To summarize the aforementioned procedure, Algorithm 1 outlines the complete workflow of the proposed representation-aware CP strategy.

### 2.4 Evaluation metrics

We evaluate affinity prediction and interval quality separately. For affinity regression, we utilize mean squared error (MSE), concordance index (CI), the squared correlation coefficient $\left(r_{m}^{2}\right)$, Pearson correlation coefficient, and Spearman correlation coefficient. Detailed formulas for these metrics are provided in Material 1.2, available as supplementary data at *Bioinformatics* online.

To assess the reliability and efficiency of the CP intervals at a significance level α, we define three key metrics. Empirical coverage measures the empirical reliability by calculating the proportion of true labels $y_{i}$ captured within the interval $\left[L_{i},U_{i}\right]$:


(24) Coverage=1N∑i=1NI(yi∈[Li,Ui])

where I(·) denotes the indicator function. Average interval width evaluates predictive efficiency, where narrower widths indicate more informative uncertainty estimates:


(25) Width=1N∑i=1N(Ui-Li)

Finally, the Average Interval Score (Winkler 1972) provides a composite evaluation by penalizing both excessive width and coverage violations:


(26) S(yi)=Ui-Li+2α(Li-yi)I(yi<Li)+2α(yi-Ui)I(yi>Ui),

(27) Average Interval Score=1N∑i=1NSi.

Lower values indicate better performance, reflecting both narrow intervals and fewer coverage violations.

Algorithm 1: Representation-aware conformal prediction (RCP)

**Input:** Pretrained model M (encoder φ, predictor f); Calibration set$\;S_{\mathrm{cal}}={\left\{(x_{i},\;y_{i})\right\}}_{i=1}^{N_{\mathrm{cal}}}$; Test sample $x_{\mathrm{test}}$; Significance level α∈(0,1); Neighborhood ratio ρ ∈ (0,1];

**Output:** Prediction interval ${\hat{C}}_{\alpha }(x_{\mathrm{test}})$ with empirical coverage at confidence level (1-α).

//**Step 1**: Feature-Residual Bank Construction

1: for each $(x_{i},\;y_{i})\;\in \;S_{\mathrm{cal}}$  **do** 

2: $\;z_{i}\;\leftarrow \varphi (x_{i}),\;{\hat{y}}_{i}\;\leftarrow \;f(z_{i})$

3: $\;\varepsilon_{i}\leftarrow \;|y_{i}-{\hat{y}}_{i}|$ //Compute nonconformity score

4: $\;{\tilde{z}}_{i}\;\leftarrow \;z_{i}/{\left||z_{i}|\right|}_{2}$ *//*$L_{2}$ normalization

5: end for

//**Step 2**: Local Calibration Set Construction

6: $z_{\mathrm{test}}\;\leftarrow \;\varphi (x_{\mathrm{test}})$, ${\tilde{z}}_{\mathrm{test}}\;\leftarrow \;z_{\mathrm{test}}/{\left||z_{\mathrm{test}}|\right|}_{2}$

7: $K\;\leftarrow \;\max(1,\;\lfloor N_{\mathrm{cal}}\cdot \rho \rfloor )$

8: $N_{K}(x_{\mathrm{test}})\;\leftarrow \;\mathrm{KNN}({\tilde{z}}_{\mathrm{test}},{\left\{{\tilde{Z}}_{i}\right\}}_{i=1}^{N_{\mathrm{cal}}})$//Retrieve indices of *K* nearest neighbors

//**Step 3**: Interval Calibration

9: ${\hat{q}}_{\mathrm{test}}\;\leftarrow \;\mathrm{Quantile}({{\{\varepsilon }_{i}\}}_{i\in N_{K}(x_{\mathrm{test}})},\;1-\alpha )$

10: ${\hat{C}}_{\mathrm{test}}\;\leftarrow \;f(z_{\mathrm{test}})$

11: ${\hat{C}}_{\alpha }(x_{\mathrm{test}})\;\leftarrow \;[{\hat{y}}_{\mathrm{test}}-{\hat{q}}_{\mathrm{test}},{\hat{y}}_{\mathrm{test}}+{\hat{q}}_{\mathrm{test}}]$

12: **return**  ${\hat{C}}_{\alpha }(x_{\mathrm{test}})$

### 2.5 Experimental settings

Our reproduced experiments use five independent random seeds, and results are summarized as mean ± SD. RCP-DTA is trained for up to 300 epochs with early stopping (patience 40). Published reference values in Table 2, available as supplementary data at *Bioinformatics* online, retain their original reporting format. Hyperparameters and computing resources are described in Material 1.3 and Table 5, available as supplementary data at *Bioinformatics* online.

## 3 Results

### 3.1 Performance evaluation on DTA prediction

We compare RCP-DTA with nine baseline methods: DeepDTA (Öztürk *et al.* 2018), GraphDTA (Nguyen *et al.* 2021), AttentionDTA (Zhao *et al.* 2023), 3DProtDTA (Voitsitskyi *et al.* 2023), PocketDTA (Zhao *et al.* 2024), ColdstartCPI (Zhao *et al.* 2025), PMMR (Ouyang *et al.* 2025), MambaTransDTA (Lou *et al.* 2026), and TSEDTA (Sun *et al.* 2026). ColdstartCPI was developed for interaction classification; our affinity-regression adaptation is denoted ColdstartCPI(Reg). The baseline results in Table 1 were obtained by retraining on the same dataset partitions as RCP-DTA, using the reported model configurations. Overall, RCP-DTA achieves competitive predictive performance across both benchmark datasets. On the Davis dataset, it slightly reduces the MSE (0.192–0.190) and improves CI from 0.900 to 0.902 compared to the strongest baseline. Notably, $r_{m}^{2}$ increases from 0.701 to 0.728, indicating improved predictive robustness. On the larger and more heterogeneous KIBA dataset, RCP-DTA shows statistically significant improvements over existing models. Specifically, it reduces the MSE by 11.465% and improves CI from 0.879 to 0.887 compared to the strongest baseline. The gains extend to correlation metrics as well.

### 3.2 Ablation study

We ran ablation experiments on the Davis and KIBA benchmarks to assess the contribution of each architectural component in RCP-DTA. As Fig. 2 shows, removing the graph encoder (w/o Stru) caused the largest drop in performance. The effect was especially stark on KIBA, where MSE increased by 0.150 (0.139–0.289), underscoring the importance of graph-based topological modeling for accurate affinity regression. Omitting the pretrained sequence embeddings (w/o Seq) similarly degraded predictive capability: on Davis, the concordance index fell by 1.663%, a decline that highlights the value of pretrained representations in capturing long-range biochemical dependencies.

**Figure 2:**
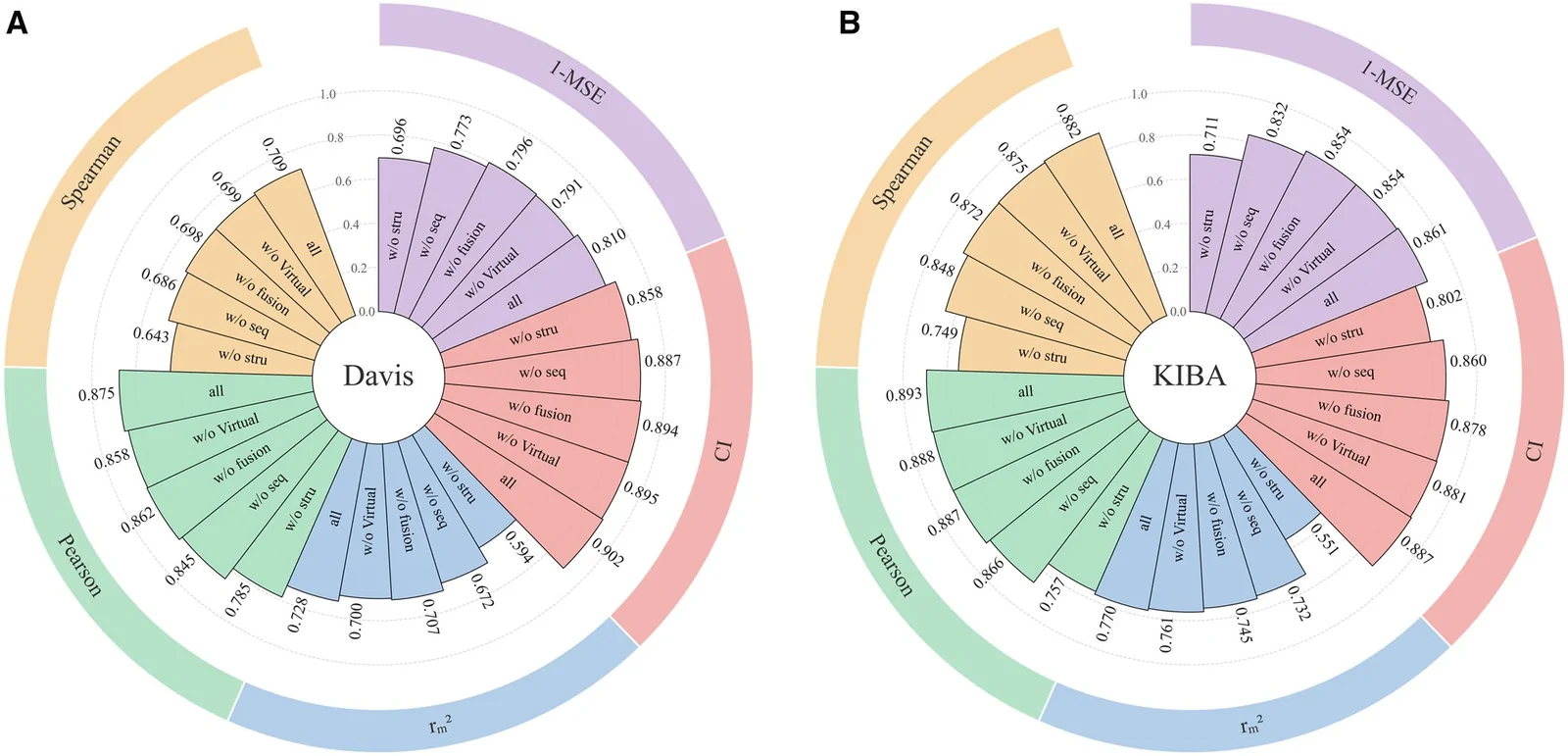
Performance contribution of individual modules in RCP-DTA on Davis and KIBA datasets. (A) Ablation results on the Davis dataset. (B) Ablation results on the KIBA dataset.

The integration and refinement mechanisms proved equally important. Replacing the gated fusion module with simple concatenation (w/o fusion) hurt performance across all metrics. On KIBA, ${r_{m}}^{2}\;$dropped by 3.247%, suggesting that linear aggregation cannot adequately reconcile the heterogeneity between sequence semantics and spatial geometries. Disabling the virtual pocket feedback mechanism (w/o Virtual) further reduced accuracy. The MSE increased by 0.019 (0.190–0.209) on Davis and by 0.007 (0.139–0.146) on KIBA. This confirmed that modeling of the local binding environment yields gains in predictive accuracy. The selection of top-3 pockets was guided by the comparative evaluation of different pocket numbers in Table 6, available as supplementary data at *Bioinformatics* online. These results indicate that RCP-DTA performs best when all components work together.

### 3.3 Generalization on cold settings

The generalization capabilities of the models against unseen entities are assessed through three realistic cold-start scenarios on the KIBA dataset (Table 2). In the unseen drugs scenario, RCP-DTA has the lowest reported mean MSE (0.365) and the highest mean CI (0.772), Pearson correlation (0.707), and Spearman correlation (0.681). ColdstartCPI(Reg) has the highest mean ${r_{m}}^{2}$ (0.445, compared with 0.438 for RCP-DTA).

Under the unseen targets setting, RCP-DTA has the highest mean CI (0.734), ${r_{m}}^{2}$ (0.425), and Spearman correlation (0.585), whereas 3DProtDTA has the lowest mean MSE (0.370 versus 0.375 for RCP-DTA), and ColdstartCPI(Reg) has the highest mean Pearson correlation (0.702 versus 0.682). Under dual cold start, RCP-DTA has the best reported mean for all five metrics. Relative to ColdstartCPI(Reg), MSE decreases from 0.582 to 0.542 (∼6.87%), and CI increases from 0.633 to 0.648. These KIBA experiments support competitive cold-start regression performance against the evaluated methods.

### 3.4 Manifold optimization and local homogeneity

We visualized the learned joint embeddings $Z_{\mathrm{complex}}$ with t-SNE (van der Maaten *et al.* 2008) to inspect the structure of the representation space. Before training, the embeddings are scattered with no discernible organization (Fig. 3A and C). After training, a clearer local structure emerges (Fig. 3B and D): samples with similar binding affinities cluster in nearby regions. To move beyond visual inspection, we quantified local consistency with a coefficient $R_{\mathrm{local}}$, defined as the ratio of neighborhood-level variance to global variance:


(28) Rlocal=1N∑i=1NVar(yj|j∈NK(i))Var(yglobal).

where $N_{K}(i)$ denotes the *K*-nearest neighbors of sample i in the learned representation space. Lower $R_{\mathrm{local}}$ values correspond to tighter affinity homogeneity within local neighborhoods.

**Figure 3:**
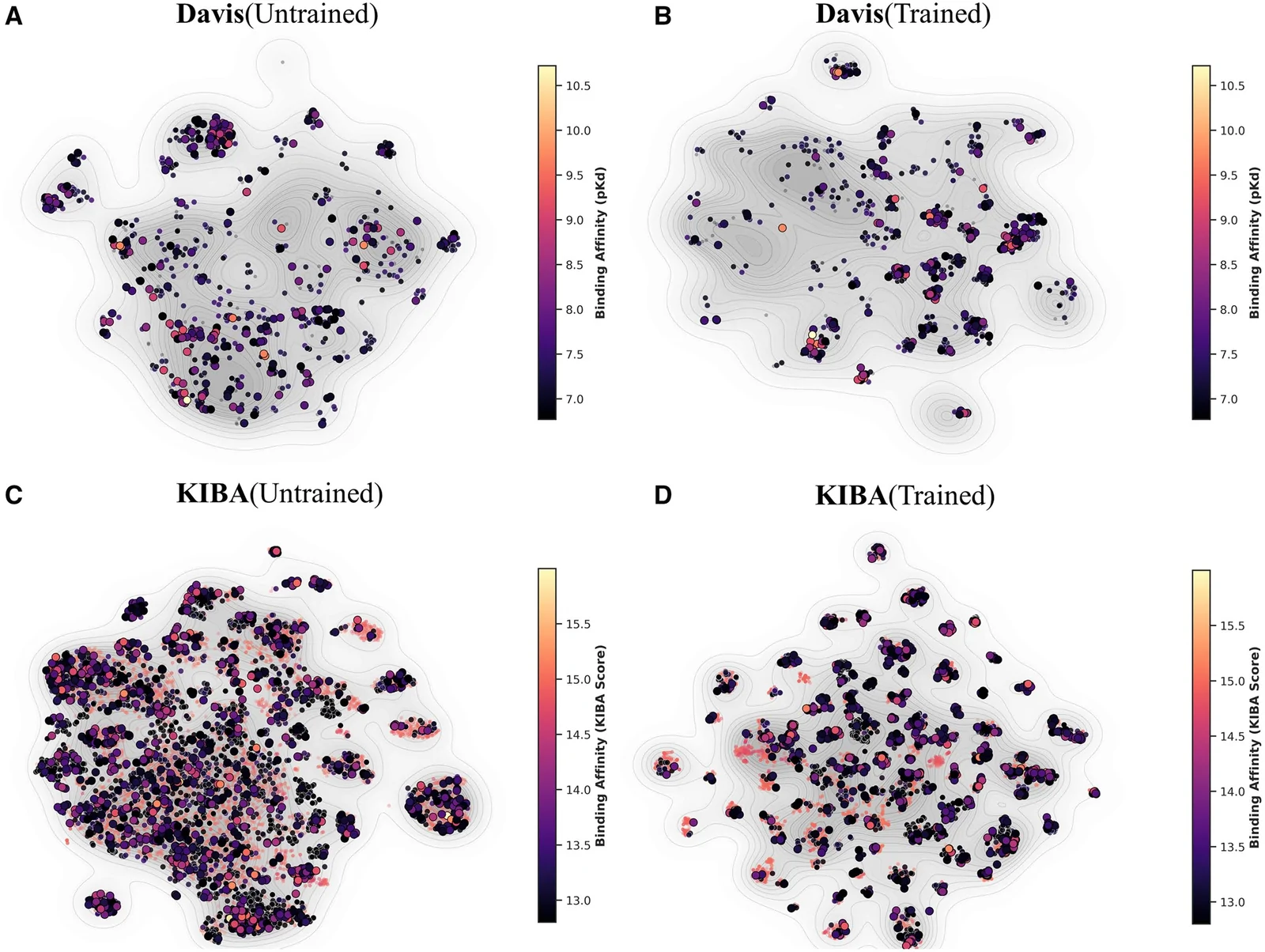
t-SNE projections of the learned embeddings on Davis and KIBA, colored by binding affinity. (A, C) Before training. (B, D) After training. Contours indicate feature density.

On Davis, $R_{\mathrm{local}}$ fell from 0.9643 to 0.8451 (a 12.361% reduction), and on KIBA from 0.9039 to 0.7698 (14.836%). For RCP, the method relies on the assumption that neighboring samples behave similarly, and a tighter neighborhood makes this assumption more plausible.

### 3.5 Reliable uncertainty quantification via RCP

Standard split CP produces intervals of constant width, as the calibration step computes a single, nonadaptive correction term from the holdout data (Oliveira *et al.* 2024). Conformalized quantile regression (CQR) allows interval width to vary with the input, but its final calibration adds a constant correction that can wash out much of this adaptivity when the initial quantile estimates are noisy (Romano *et al.* 2019). To further evaluate the effectiveness of representation-aware local calibration, we additionally compared RCP-DTA with the Dynamic Applicability Domain (dAD) method (Oršolić and Šmuc 2023), a local calibration strategy that constructs sample-specific calibration domains based on predefined similarity.

Table 3 summarizes the calibration results. While dAD improves locality by selecting calibration samples based on structural similarity, it tends to generate conservative prediction intervals with substantially increased widths. This suggests that predefined similarity-based domains may provide reliable coverage at the cost of interval efficiency in heterogeneous DTA tasks. In contrast, RCP-DTA consistently achieves empirical coverage close to nominal levels while improving interval efficiency. The effect of neighborhood proportion *ρ* on interval efficiency is analyzed in Fig. 2, available as supplementary data at *Bioinformatics* online, and the corresponding inference time under different *ρ* settings is summarized in Table 7, available as supplementary data at *Bioinformatics* online. Full results under cold-start settings are provided in Material 2.1 and Tables 8–10, available as supplementary data at *Bioinformatics* online.

Fig. 4 examines how prediction intervals respond to varying error levels. CP and CQR produce constant interval widths regardless of prediction error (Fig. 4A and B). In low-error regions, RCP yields substantially tighter intervals than both baselines. As error grows, intervals widen and eventually surpass those of CP and CQR in high-error regions. This pattern indicates that RCP adapts its uncertainty estimates to the underlying error magnitude rather than applying a fixed margin. As shown in Fig. 4C and D, prediction error and interval width are positively correlated on both datasets, with the association reaching statistical significance. This adaptive behavior makes the intervals more interpretable. A narrow interval signals a confident prediction, while a wide one flags uncertainty that a screening chemist would want to scrutinize further. Further stratified analysis across different binding affinity levels is provided in Fig. 3, available as supplementary data at *Bioinformatics* online.

**Figure 4:**
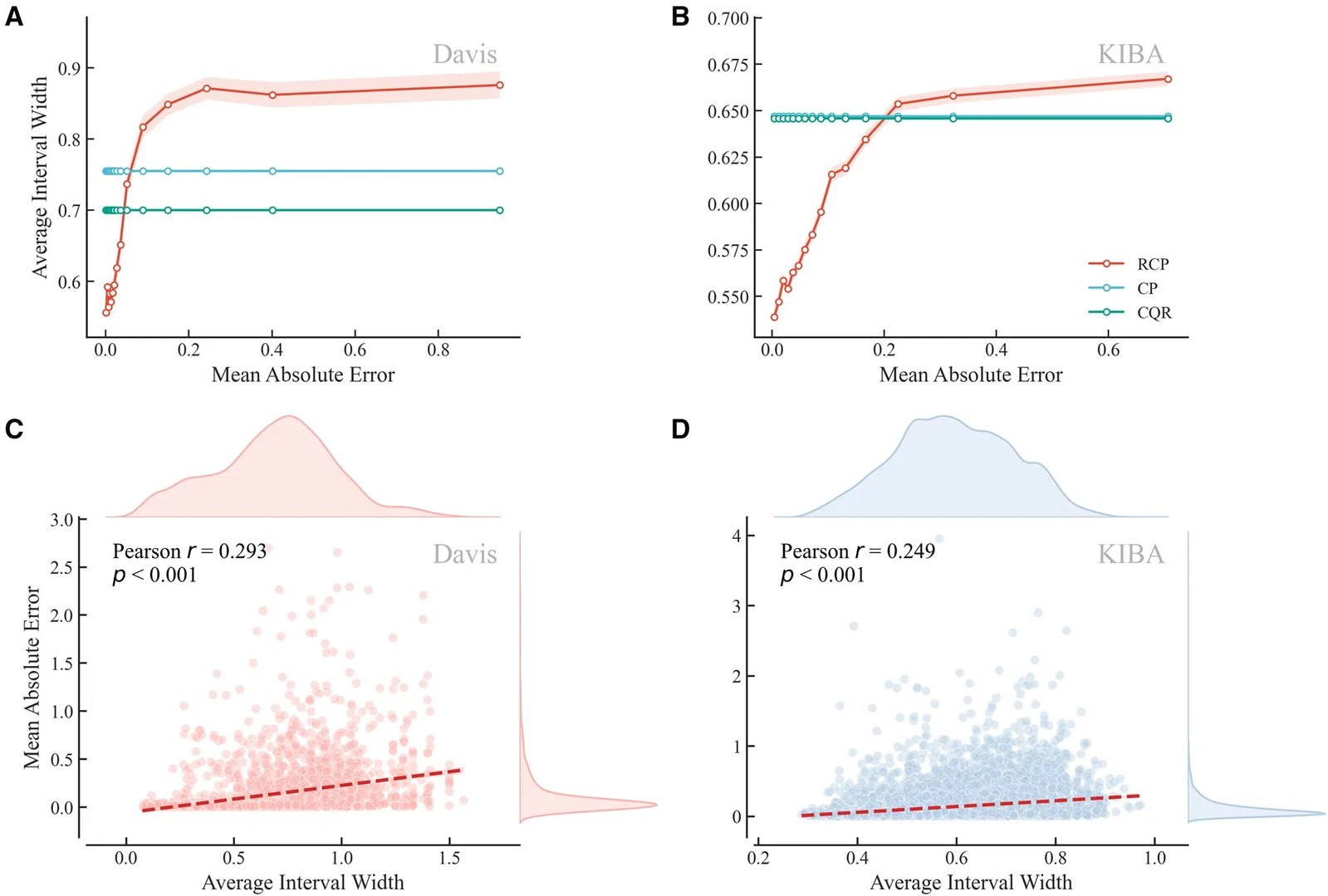
Uncertainty calibration and correlation analysis of the prediction intervals on the Davis and KIBA datasets. (A, B) Comparison of average interval widths across varying MAE levels for RCP, CP, and CQR methods. (C, D) Joint distribution of MAE and average interval width for RCP.

### 3.6 Model-agnosticism and generality of the RCP framework

The cross-architecture adaptability of the RCP framework is evaluated by integrating it as a plug-and-play module into multiple backbone networks. Results on the Davis and KIBA benchmarks with the neighborhood ratio fixed at *ρ* = 0.1 are presented in Fig. 5. As shown in Fig. 5A, the empirical coverage at significance level α=0.1 remains close to the nominal target across most backbone models. Meanwhile, substantial differences in interval efficiency were observed among models (Fig. 5B). RCP-DTA consistently achieved the narrowest prediction intervals across all evaluated backbones, indicating improved uncertainty efficiency while maintaining reliable coverage. Overall, these results demonstrate that the RCP framework provides architecture-agnostic uncertainty calibration. Additional evaluations under OOD scenarios are provided in Fig. 4, available as supplementary data at *Bioinformatics* online.

**Figure 5:**
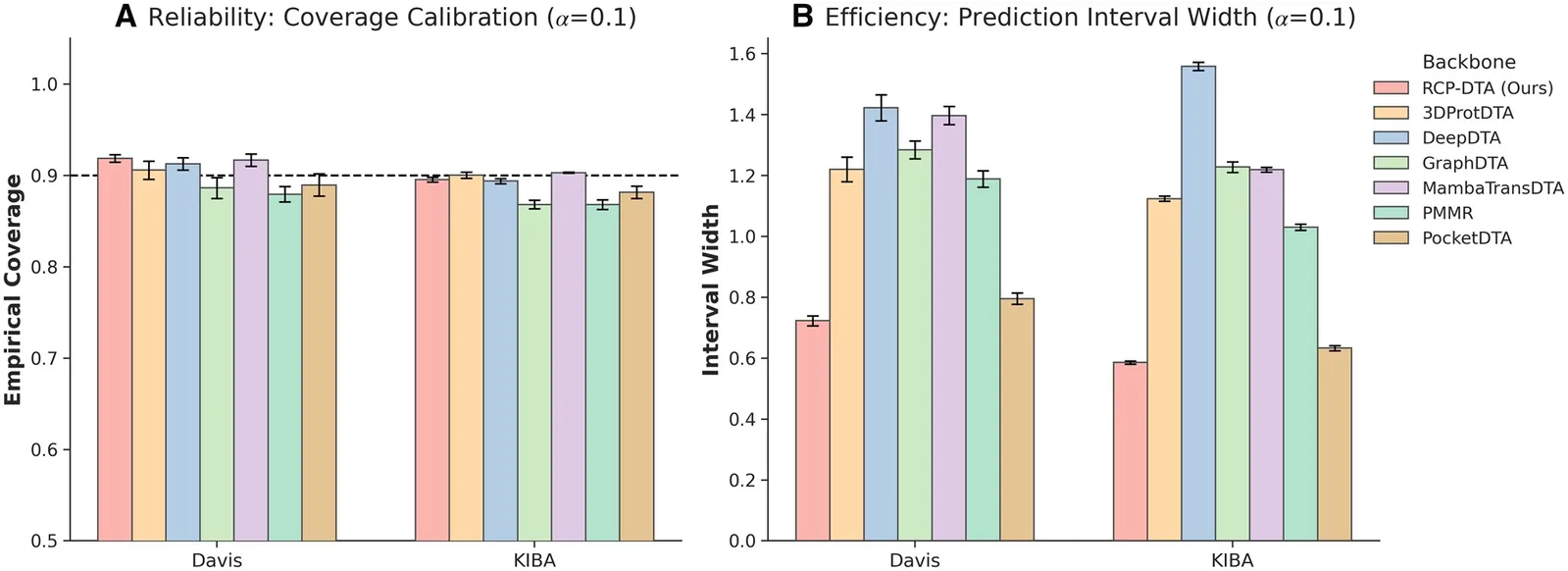
Reliability and efficiency evaluation of the RCP framework across diverse backbone architectures. (A) Empirical coverage calibration across standard scenarios at α=0.1. (B) Average prediction interval width for the corresponding models.

### 3.7 Case study

The adaptive behavior of RCP in unseen chemical space is further examined through two clinically relevant kinase targets (Patnaik *et al.* 2022): EGFR (ErbB1) and HER2 (ErbB2). Models are evaluated under the unseen-drug setting. Fig. 6 presents the predicted affinities together with RCP and CQR prediction intervals. For the EGFR target (Fig. 6A, N=107), the model yields an RMSE of 0.372 and an empirical coverage of 88.8%. For the HER2 target (Fig. 6B, N=68), the model achieves a lower RMSE of 0.224 and a coverage of 92.6%. Compared with CQR, RCP produced consistently narrower intervals at comparable coverage, yielding more efficient uncertainty estimates.

**Figure 6:**
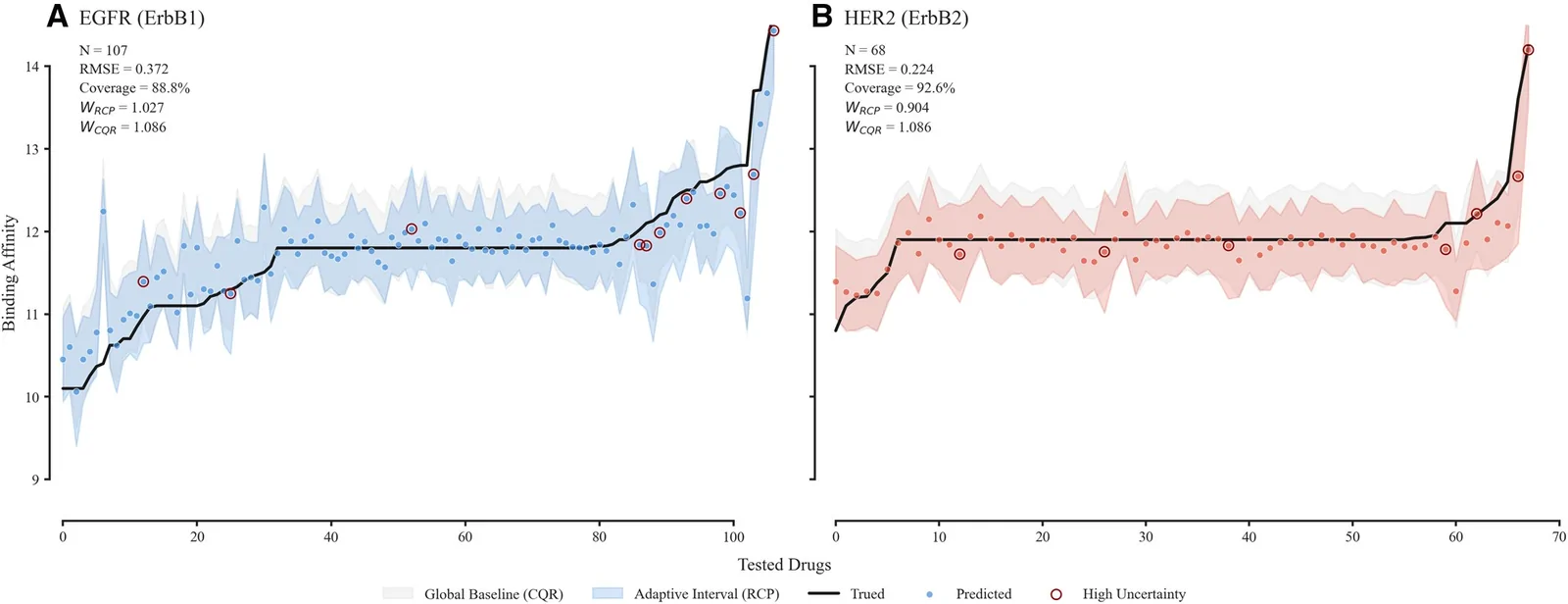
Prediction intervals for individual drugs targeting EGFR (A) and HER2 (B) in the cold-drug setting. Drugs are sorted by true binding affinity.

To further examine this behavior, Fig. 7 highlights representative high and low uncertainty cases. For EGFR, the high-uncertainty sample (Fig. 7A) exhibits a wide interval (W=1.72) and a large prediction error (Err=1.01), whereas the low-uncertainty sample (Fig. 7B) shows a tight interval (W=0.66) with a small error (Err=0.17). A similar pattern is observed for HER2. We further compare this behavior with structural similarity to the training set, measured by the maximum Tanimoto similarity ${(\mathrm{TS}}_{\max})$. Similarity alone is a poor proxy for predictive difficulty. In Fig. 7A, the sample with the largest uncertainty carries a fairly high Tanimoto score ${(\mathrm{TS}}_{\max}=0.73)$ but nonetheless showed substantial error. In Fig. 7B, a sample with low similarity ${(\mathrm{TS}}_{\max}=0.28)$ is predicted with little error. What these cases suggest is that uncertainty measured in the learned representation space tracks prediction confidence more faithfully than 2D similarity does, especially in regions of chemical space the model has not seen during training. Additional structural analysis of a representative low-uncertainty complex provides supporting evidence that the Virtual Pocket Feedback mechanism captures biologically relevant binding regions (Fig. 5, available as supplementary data at *Bioinformatics* online).

**Figure 7:**
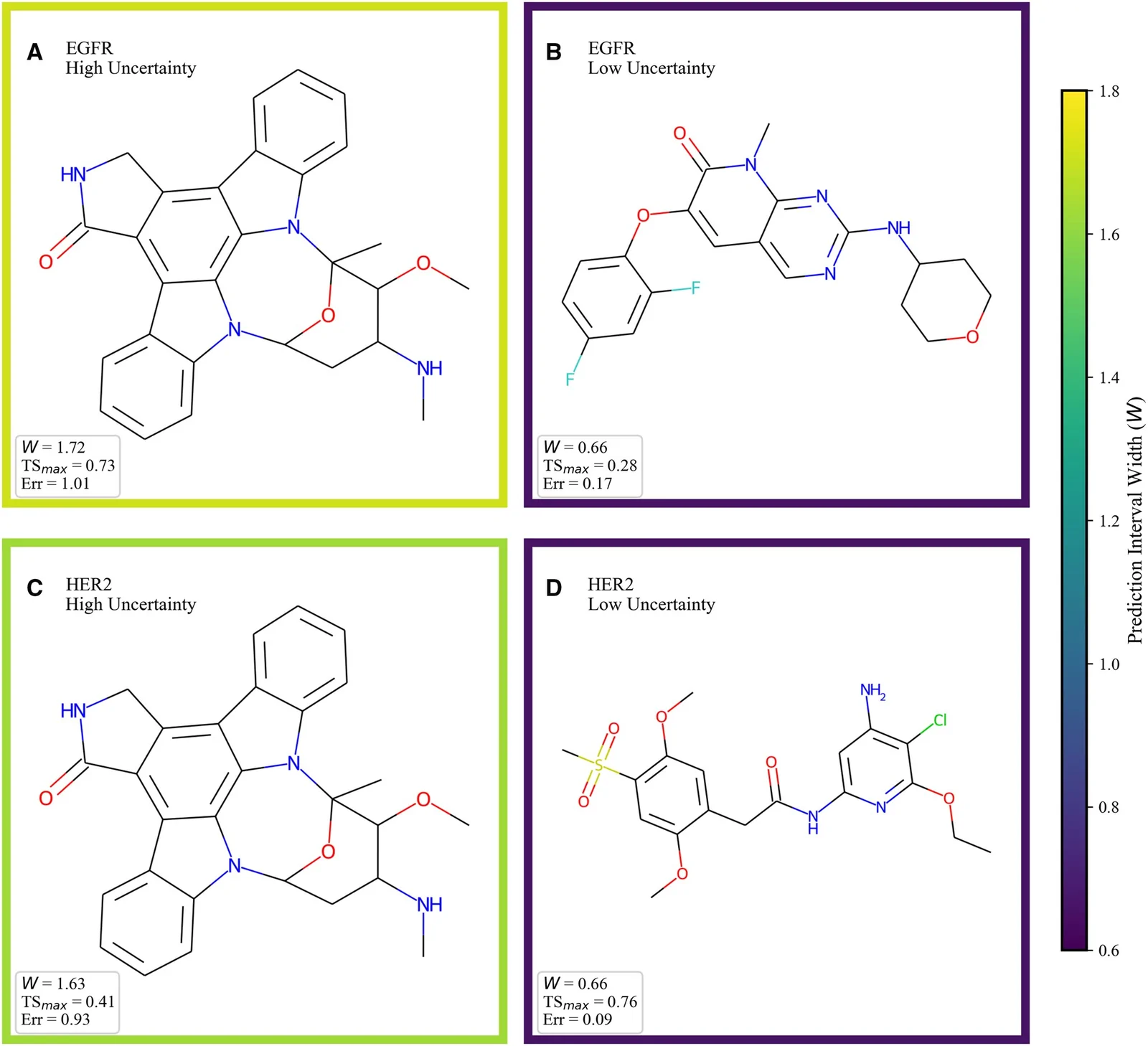
Representative molecular cases exhibiting high and low prediction uncertainty for the EGFR and HER2 targets. (A) EGFR with high uncertainty. (B) EGFR with low uncertainty. (C) HER2 with high uncertainty. (D) HER2 with low uncertainty.

## 4 Conclusion

We introduced RCP-DTA, a representation-aware framework that integrates DTA prediction with reliable uncertainty quantification. The model combines sequence-based semantic features with graph representations of binding pockets and, on standard benchmarks, exceeds the predictive accuracy of existing methods. Its uncertainty estimates come from the RCP mechanism, which conditions prediction intervals on the local structure of the learned representation space rather than relying on a global calibration rule. This calibration remained stable under cold-start splits, where training and test distributions differ sharply. On the practical side, RCP-DTA produces a prediction interval at a user-specified confidence level. Compounds with high predicted affinity and narrow intervals can be prioritized as strong candidates, while wide intervals flag compounds that need experimental verification. Despite these advantages, several limitations remain. Reliance on predicted protein structures and pockets may introduce noise that compromises model performance. Consequently, the downstream uncertainty estimates may be inherently affected by upstream structural inaccuracies, low-confidence regions, or proteins exhibiting significant conformational flexibility. Future iterations of the RCP framework could mitigate these issues by incorporating confidence-weighted structural representations or utilizing ensemble structures derived from molecular dynamics to capture conformational sampling. In addition, while RCP ensures marginal coverage, achieving conditional coverage under distribution shifts remains an open challenge. In future research, we will explore more robust representation learning and adaptive calibration strategies to improve reliability under diverse and evolving data distributions.

## Supplementary Material

btag771_Supplementary_Data
