# Supplementary material for: Representation-aware conformal prediction for reliable uncertainty quantification in drug-target affinity
Source: Bioinformatics. 2026 Oct 13;42(10):btag771. doi: 10.1093/bioinformatics/btag771 (PMC13719223; doi:10.1093/bioinformatics/btag771)
Supplement: Supplementary file 1 [file btag771_supplementary_data.pdf]

Supplementary Material

Representation-aware conformal prediction for reliable uncertainty quantification in drug-target affinity

*Xinya Li, Yaxin Zhang, Ximin Zeng, Danping Zhou, Yue Cheng, Shaoping Shi**

Department of Mathematics, School of Mathematics and Computer Sciences, Nanchang University, Nanchang 330031, China

*Corresponding author. Department of Mathematics, School of Mathematics and Computer Sciences, Nanchang University, Nanchang 330031, China.

E-mail: shishaoping@ncu.edu.cn

Contents

1 Materials and Methods

1.1 Benchmark datasets

1.2 Evaluation metrics

1.3 Experiment setting

2 Results

2.1 Comprehensive performance evaluation under Out-of-Distribution scenarios

3 Supplementary Tables

Table S1. Detailed statistics of the processed benchmarking datasets.

Table S2. Performance comparison of RCP-DTA and SOTA models on Metz and BindingDB datasets.

Table S3. Atom and bond feature specifications for drug molecular graph construction.

Table S4. Specification of node-level features for protein geometric graph representations.

Table S5. Hyperparameter search space and optimal configurations for RCP-DTA.

Table S6. Sensitivity analysis of the number of retained binding pockets.

Table S7. Inference latency comparison between standard CP and RCP-DTA with different neighborhood ratios.

Table S8. Comparison of global and localized conformal prediction methods under the unseen drugs setting on KIBA dataset.

Table S9. Comparison of global and localized conformal prediction methods under the unseen targets setting on KIBA dataset.

Table S10. Comparison of global and localized conformal prediction methods under the dual cold-start setting on KIBA dataset.

4 Supplementary Figures

Figure S1. Statistical distribution of structural confidence scores across distinct protein regions.

Figure S2. Sensitivity analysis of neighborhood ratio ρ on coverage, width, and interval score.

Figure S3. Reliability and efficiency analysis across different binding affinity levels.

Figure S4. Reliability and efficiency evaluation of the RCP framework across diverse backbone architectures.

Figure S5. Structural validation and interpretability analysis of the EGFR-ligand interaction in Pocket-1.

References

1 Materials and methods

1.1 Benchmark datasets

The Davis Dataset (Davis et al. 2011) comprises interactions between 442 kinases and 68 inhibitors. The binding affinity was originally measured by the equilibrium dissociation constant K_d. Following standard protocols in the DTA field, K_d values were transformed (as shown in Equation (1)) into logarithmic scale pK_d . For the KIBA dataset (Tang et al. 2014), we utilized the preprocessed version provided by PocketDTA (Zhao et al. 2024), which integrates K_i, K_d, and IC_{50} values while excluding low-confidence interactions to ensure cross-experimental consistency. The final KIBA dataset used for the experiment includes 228 proteins and 2,061 ligands, comprising a total of 117,395 valid interaction pairs. The Metz (Metz et al. 2011) dataset contains experimentally measured binding affinities between drugs and target proteins, while BindingDB (Gilson et al. 2016) is a large-scale public database containing diverse protein-ligand interaction records collected from multiple sources. The processed datasets and corresponding benchmark protocols were obtained from the TSEDTA (Sun et al. 2026) study.

$$pK_d = -\log_{10}\left(\frac{K_d}{1e^9}\right) \tag{1}$$

To construct the multimodal features, we implement an automated structure acquisition pipeline. We retrieve the three-dimensional conformations of target kinases from the AlphaFold Database (v6) (Varadi et al. 2024). For sequences absent from the database, we use ColabFold (v1.6.1) (Mirdita et al. 2022) for de novo structure prediction, retaining only the conformation with the highest average pLDDT score to

ensure structural credibility. The P2Rank (v2.4) (Kriválk and Hoksza 2018) algorithm then predicts ligand binding sites on the acquired structures to enable the virtual pocket feedback mechanism. We extract the top three potential pocket centers based on their prediction scores to cover true binding pockets while minimizing background noise.

1.2 Evaluation metrics

To evaluate the predictive performance of the models, we employed several standard metrics. Mean Squared Error (MSE) measures the average squared deviation between the predicted values ($\hat{y}_i$) and the ground-truth values (y_i):

$$MSE = \frac{1}{N}\sum_{i=1}^{N}(y_i - \hat{y}_i)^2 \tag{2}$$

where N denotes the total number of samples.

Concordance Index (CI) is utilized to assess the model's ranking accuracy by evaluating whether the predicted values preserve the relative order of the true values:

$$CI = \frac{1}{Z}\sum_{y_i > y_j} h\left(\hat{y}_i - \hat{y}_j\right) \tag{3}$$

where Z is a normalization constant (the number of pairs with different labels), and $h(x)$ is the step function:

$$h(x) = \begin{cases} 1 & x > 0 \\ 0.5 & x = 0 \\ 0 & x < 0 \end{cases} \tag{4}$$

Pearson correlation coefficient (Pearson) measures the degree of linear correlation between the predicted and true values:

$$\text{Pearson} = \frac{\sum_{i=1}^{N}(y_i - \bar{y})\left(\hat{y}_i - \bar{\hat{y}}\right)}{\sqrt{\sum_{i=1}^{N}(y_i - \bar{y})^2}\sqrt{\sum_{i=1}^{N}\left(\hat{y}_i - \bar{\hat{y}}\right)^2}} \tag{5}$$

where $\bar{y}$ and $\bar{\hat{y}}$ denote the means of the true and predicted values, respectively.

Coefficient of Determination (r_m^2) is used to further assess the external predictive power of the regression model:

$$r_m^2 = r^2\left(1 - \sqrt{(r^2 - r_0^2)}\right) \tag{6}$$

where r_0^2 denotes the squared correlation coefficient of the linear regression between predicted and true values forced through the origin.

Spearman Rank Correlation Coefficient $(Spearman)$ evaluates the monotonic relationship between variables by calculating the Pearson correlation between their ranks:

$$Spearman = 1 - \frac{6\sum d_i^2}{N(N^2-1)} \tag{7}$$

where d_i represents the difference between the ranks of the true value and the predicted value for the i-th sample.

1.3 Experiment setting

The model was optimized using the RAdam (Liu et al. 2019) algorithm coupled with a Lookahead (Zhang et al. 2019) mechanism to stabilize convergence and escape local optima. The Lookahead mechanism was configured with a synchronization period of 5 steps and a linear interpolation factor $\beta = 0.5$. For the cosine annealing schedule (Loshchilov and Hutter 2016), the initial period was set to $T_0 = 10$, the period multiplier to $T_{mult} = 2$, and the minimum learning rate to $\eta_{min} = 1 \times 10^{-5}$. These parameters were optimized to balance convergence speed and global search capability. All deep learning models in this study were implemented and evaluated on a Linux-based server. The specific hardware and software configurations are summarized as follows: Training tasks were deployed on a high-performance computing cluster

equipped with an Intel Xeon Gold 5318Y CPU (2.1 GHz) and an NVIDIA A800 (80GB) PCIe GPU. The framework was implemented using Python 3.7.12 and PyTorch 1.13.1 (Paszke et al. 2019). CUDA was employed for GPU-accelerated computation to ensure efficiency.

2 Results

2.1 Comprehensive performance evaluation under Out-of-Distribution scenarios

To evaluate the generalization and adaptability of the RCP framework, we conducted extensive experiments under three stringent Out-of-Distribution settings: Unseen Drug (Table S8), Unseen Target (Table S9), and Dual Cold-start (Table S10).

In the Unseen Drug and Unseen Target settings, the RCP variants demonstrate exceptional predictive efficiency while strictly adhering to the theoretical coverage requirements. For the Unseen Drug scenario at $\alpha = 0.1$, RCP_0.10 achieves a highly competitive interval width of 0.929±0.006, which is significantly narrower than the 1.026±0.017 produced by the standard CP method. Similarly, in the Unseen Target setting, RCP maintains superior Score values (e.g., 2.031±0.029 for RCP_0.05 at $\alpha = 0.05$), indicating that by leveraging local representation-aware density, the model can effectively compress prediction intervals without sacrificing statistical reliability. This suggests that when at least one side of the drug-target pair is well-characterized in the representation space, RCP can capitalize on local data characteristics to provide highly precise confidence bounds. The Dual Cold-start scenario represents the most rigorous test, where neither the drug nor the target has been encountered during training. The reduced empirical coverage in the dual cold-start setting mainly results from the severe

distribution shift introduced by simultaneously unseen drugs and targets. Although the retrieved neighbors are selected from the learned representation space, their residual distributions may become less homogeneous when the test samples are located far from the training distribution.

RCP-DTA provides empirically calibrated and adaptive uncertainty estimation within the learned representation space, rather than guaranteeing perfect coverage under arbitrary out-of-distribution scenarios. Future work will investigate incorporating explicit OOD detection, uncertainty-aware neighborhood retrieval, and adaptive calibration strategies for samples beyond the training distribution.

3 Supplementary Tables

Table S1. Detailed statistics of the processed benchmarking datasets.

Dataset	Drugs	Target	Affinity Range	Total
Davis	68	442	[5.00,10.80]	30,056
KIBA	2061	228	[0.00,17.20]	117,395
Metz	1423	170	[4.00,11.10]	35,259
BindingDB	9860	1088	[2.00,14.00]	42,201

We evaluated RCP-DTA on Metz and BindingDB using an 8:1:1 training/validation/test protocol. These experiments assess point prediction and do not use a separate calibration subset. Table S2 combines our reproduced results with published reference values reported in TSEDTA (Sun et al. 2026). The reproduced AttentionDTA, 3DProtDTA, PMMR, and RCP-DTA results use the same split and evaluation protocol within each dataset. Published reference rows are retained for context; identical split proportions alone do not establish identical sample partitions or preprocessing across studies.

Table S2. Performance comparison of RCP-DTA and SOTA models on Metz and BindingDB datasets.

Dataset	Model	MSE↓	CI↑	r_m^2↑	Pearson↑	Spearman↑
Metz	DeepDTA[a]	0.353	0.703	0.537	-	-
	GraphDTA[a]	0.317	0.801	0.620	-	-
	modality-DTA[a]	**0.281**	0.794	-	-	-
	AttentionDTA[b]	0.302±0.006	0.803±0.008	0.645±0.011	0.818±0.013	0.771±0.017
	3DProtDTA[b]	0.286±0.005	0.811±0.010	**0.669±0.020**	0.817±0.015	0.784±0.020
	MambaTransDTA[a]	0.302	0.804	0.636	-	-
	PMMR[b]	0.311±0.005	0.808±0.007	0.633±0.013	0.804±0.008	0.762±0.015
	TSEDTA[a]	0.3346	0.7955	0.571	-	-
	**RCP-DTA**	**0.281±0.007**	**0.816±0.006**	0.650±0.014	**0.840±0.009**	**0.792±0.012**
BindingDB	KronRLS[a]	0.939	0.815	-	-	-
	DeepDTA[a]	0.703	0.826	0.669	-	-
	DeepCDA[a]	0.808	0.822	0.631	-	-
	GraphDTA[a]	0.593	0.855	0.682	-	-
	AttentionDTA[b]	0.482±0.024	0.865±0.007	0.706±0.009	0.866±0.004	0.796±0.013
	3DProtDTA[b]	0.480±0.013	0.852±0.011	0.705±0.005	0.860±0.002	**0.806±0.007**
	DoubleSG-DTA[a]	0.533	0.862	**0.726**	-	-
	ELECTRA-DTA[a]	0.650	0.837	0.670	-	-
	MambaTransDTA[a]	0.715	0.817	0.637	-	-
	PMMR[b]	0.529±0.014	0.829±0.008	0.689±0.007	0.834±0.004	0.791±0.006
	TSEDTA[a]	0.5018	**0.8698**	0.7018	-	-
	**RCP-DTA**	**0.472±0.010**	0.869±0.004	0.698±0.030	**0.868±0.003**	0.801±0.004

Bold values indicate the best performance. Superscript a denotes values taken from the benchmark reported in TSEDTA; superscript b denotes values obtained from our reproduction experiments under the same data split and evaluation protocol.

Table S3. Atom and bond feature specifications for drug molecular graph construction.

Feature type	Feature Description	Dimension	Encoding Method
Drug Atom	Atomic element (C, N, O, S, F, P, Cl, Br, B, H, etc.)	44	One-hot
	Atomic degree (Number of covalently bonded neighbors)	11	One-hot
	Total number of hydrogen atoms	11	One-hot
	Implicit valence	11	One-hot
	Aromaticity (Whether the atom belongs to an aromatic ring)	1	Boolean
Drug Edge	Bond type: Single	1	Boolean
	Bond type: Double	1	Boolean
	Bond type: Triple	1	Boolean
	Bond type: Aromatic	1	Boolean
	Conjugation (Whether the bond is part of a conjugated system)	1	Boolean
	Ring membership (Whether the bond is within a ring structure)	1	Boolean

Table S4. Specification of node-level features for protein geometric graph representations.

Feature Category	Feature Descriptor	Dimension	Description
Biological Identity	Amino Acid Type	21	One-hot encoding (20 standard AA + 'X' for unknown)
Sequential Context	Positional Encoding	16	Absolute sequence position encoding
Evolutionary Profile	PSSM Features	20	Log-likelihood of residue substitution (Normalized)
Local Geometry	Dihedral Angles	4	sin and cos values of backbone dihedral angles (ϕ, ψ)

To account for the periodicity of protein backbone torsion angles, the dihedral angles ϕ and ψ were encoded as ($\sin\phi$, $\cos\phi$, $\sin\psi$, $\cos\psi$), resulting in a 4-dimensional geometric descriptor for each residue.

Table S5. Hyperparameter search space and optimal configurations for RCP-DTA.

Dataset	Hyperparameter	Search range	Optimized Value
Davis	Batch size	{32,64,128,256,512}	64
	Weight decay	{0.001,0.002,0.005,0.01,0.02}	0.002
KIBA,Metz BindingDB	Batch size	{32,64,128,256,512}	128
	Weight decay	{0.001,0.002,0.005,0.01,0.02}	0.005
All	Drug latent dim (d_{drug})	{64,128,256,512}	256
	Protein latent dim (d_{pro})	{64,128,256,512}	256
	Attention heads	{1,2,4,8}	4
	Learning rate	{0.001,0.002,0.005,0.01,0.02}	0.001
	Training epochs	{150,200,250,300}	300
	Early stopping	{40,60,80}	40

Although P2Rank provides reliable pocket predictions, retaining only the highest-ranked pocket may discard potentially relevant binding regions when the true binding interface is not ranked first due to structural flexibility or prediction uncertainty. Conversely, including too many candidate pockets may introduce irrelevant spatial regions and redundant structural information, which can negatively affect representation learning.

Table S6. Sensitivity analysis of the number of retained binding pockets.

Dataset	Model	MSE↓	CI↑	r_m^2↑	Pearson↑	Spearman↑
Davis	Top-1 Pocket	0.213±0.015	0.898±0.005	0.700±0.038	0.860±0.013	0.707±0.009
	Top-3 Pocket	**0.190±0.016**	**0.902±0.003**	**0.728±0.033**	**0.875±0.007**	0.709±0.011
	Top-5 Pocket	0.207±0.008	**0.902±0.004**	0.709±0.034	0.865±0.008	**0.713±0.011**
	All Pocket	0.206±0.006	0.900±0.005	0.700±0.037	0.866±0.001	0.710±0.012
KIBA	Top-1 Pocket	0.147±0.006	0.883±0.002	0.758±0.008	0.887±0.004	0.880±0.001
	Top-3 Pocket	**0.139±0.006**	**0.887±0.002**	**0.770±0.008**	**0.893±0.004**	**0.882±0.003**
	Top-5 Pocket	0.151±0.001	0.877±0.003	0.761±0.015	0.884±0.002	0.871±0.005
	All Pocket	0.166±0.017	0.877±0.005	0.724±0.044	0.873±0.009	0.868±0.008

Bold values indicate the best performance.

Table S7. Inference latency comparison between standard CP and RCP-DTA with different neighborhood ratios.

Dataset	Model	ρ	Inference time(s)	Time/sample(ms)
Davis	Standard CP	-	2.4e-5	8.0e-6
	RCP	0.01	0.102	0.03
	RCP	0.05	0.193	0.06
	RCP	0.10	0.289	0.10
	RCP	0.50	1.105	0.37
	RCP	0.95	1.627	0.54
KIBA	Standard CP	-	2.9e-05	2.4e-6
	RCP	0.01	1.590	0.14
	RCP	0.05	3.108	0.26
	RCP	0.10	4.874	0.42
	RCP	0.50	19.719	1.68
	RCP	0.95	29.012	2.47

Table S8. Comparison of global and localized conformal prediction methods under the unseen drugs setting on the KIBA dataset.

α	Method	Coverage↑	Width↓	Score↓
0.05	CP	0.952±0.003	1.523±0.021	2.501±0.053
	CQR	0.951±0.003	1.506±0.022	2.480±0.050
	dAD(CV)	0.966±0.003	3.869±0.055	4.607±0.025
	dAD(NN)	**0.967±0.003**	3.911±0.054	4.623±0.025
	RCP_0.05	0.954±0.003	1.410±0.019	**2.216±0.050**
	RCP_0.10	0.951±0.003	**1.393±0.017**	2.299±0.056
	RCP_0.30	0.949±0.003	1.417±0.019	2.397±0.055
	RCP_0.50	0.949±0.003	1.448±0.020	2.436±0.054
	RCP_0.75	0.950±0.003	1.488±0.023	2.475±0.053
0.1	CP	0.899±0.003	1.026±0.017	1.868±0.032
	CQR	0.900±0.003	1.013±0.011	1.846±0.031
	dAD(CV)	0.929±0.005	2.800±0.047	3.708±0.024
	dAD(NN)	**0.930±0.005**	2.831±0.046	3.719±0.022
	RCP_0.05	0.899±0.002	0.943±0.005	**1.702±0.032**
	RCP_0.10	0.895±0.002	**0.929±0.006**	1.751±0.033
	RCP_0.30	0.894±0.003	0.945±0.009	1.805±0.033
	RCP_0.50	0.895±0.003	0.968±0.010	1.827±0.033
	RCP_0.75	0.897±0.003	0.996±0.010	1.849±0.033
0.2	CP	0.798±0.005	0.617±0.006	1.332±0.018
	CQR	0.799±0.003	0.629±0.005	1.319±0.017
	dAD(CV)	0.849±0.004	1.714±0.023	2.801±0.027
	dAD(NN)	**0.851±0.005**	1.745±0.023	2.814±0.028
	RCP_0.05	0.790±0.004	0.566±0.003	**1.251±0.018**
	RCP_0.10	0.786±0.004	**0.555±0.004**	1.271±0.018
	RCP_0.30	0.786±0.005	0.562±0.005	1.295±0.018
	RCP_0.50	0.787±0.005	0.575±0.005	1.307±0.018
	RCP_0.75	0.792±0.005	0.595±0.006	1.320±0.018

Bold values indicate the best performance. RCP_ρ denotes the proposed method with neighborhood proportion ρ, which determines the number of nearest neighbors used for local quantile estimation as a fraction of the calibration set.

Table S9. Comparison of global and localized conformal prediction methods under the unseen targets setting on the KIBA dataset.

α	Method	Coverage↑	Width↓	Score↓
0.05	CP	0.951±0.002	2.019±0.016	2.784±0.042
	CQR	0.951±0.002	2.025±0.016	2.778±0.041
	dAD(CV)	**0.965±0.003**	4.886±0.065	5.776±0.070
	dAD(NN)	**0.965±0.003**	4.935±0.065	5.792±0.068
	RCP_0.05	0.956±0.003	**1.817±0.014**	**2.520±0.049**
	RCP_0.10	0.956±0.003	1.894±0.009	2.611±0.048
	RCP_0.30	0.952±0.001	1.956±0.009	2.712±0.046
	RCP_0.50	0.952±0.002	1.982±0.012	2.734±0.045
	RCP_0.75	0.952±0.002	2.000±0.012	2.758±0.043
0.1	CP	0.901±0.004	1.579±0.014	2.273±0.025
	CQR	0.901±0.004	1.582±0.013	2.273±0.025
	dAD(CV)	0.927±0.002	3.617±0.035	4.708±0.043
	dAD(NN)	**0.928±0.002**	3.661±0.037	4.722±0.041
	RCP_0.05	0.909±0.004	**1.391±0.008**	**2.031±0.029**
	RCP_0.10	0.911±0.002	1.473±0.007	2.111±0.024
	RCP_0.30	0.908±0.002	1.575±0.010	2.227±0.025
	RCP_0.50	0.906±0.003	1.594±0.013	2.245±0.025
	RCP_0.75	0.905±0.004	1.587±0.013	2.255±0.024
0.2	CP	0.804±0.005	1.044±0.012	1.778±0.016
	CQR	0.803±0.005	1.041±0.011	1.777±0.016
	dAD(CV)	0.845±0.003	2.373±0.014	3.636±0.027
	dAD(NN)	**0.846±0.003**	2.408±0.015	3.647±0.029
	RCP_0.05	0.806±0.005	**0.951±0.007**	**1.565±0.019**
	RCP_0.10	0.809±0.004	0.992±0.008	1.618±0.018
	RCP_0.30	0.811±0.005	1.057±0.012	1.734±0.016
	RCP_0.50	0.813±0.006	1.083±0.014	1.762±0.016
	RCP_0.75	0.808±0.006	1.060±0.014	1.765±0.016

Bold values indicate the best performance. RCP_ρ denotes the proposed method with neighborhood proportion ρ, which determines the number of nearest neighbors used for local quantile estimation as a fraction of the calibration set.

Table S10. Comparison of global and localized conformal prediction methods under the dual cold-start setting on the KIBA dataset.

α	Method	Coverage↑	Width↓	Score↓
0.05	CP	0.946±0.008	2.437±0.077	3.447±0.186
	CQR	0.949±0.010	2.416±0.106	**3.245±0.171**
	dAD(CV)	**0.952±0.010**	4.252±0.165	5.607±0.193
	dAD(NN)	**0.952**±0.010	4.292±0.164	5.608±0.182
	RCP_0.05	0.939±0.006	2.222±0.094	3.396±0.167
	RCP_0.10	0.931±0.011	**2.095±0.069**	3.364±0.243
	RCP_0.30	0.927±0.011	2.100±0.118	3.409±0.216
	RCP_0.50	0.937±0.009	2.229±0.107	3.409±0.194
	RCP_0.75	0.942±0.007	2.327±0.058	3.440±0.199
0.1	CP	0.894±0.009	1.734±0.044	2.775±0.115
	CQR	0.896±0.008	1.784±0.061	**2.683±0.105**
	dAD(CV)	0.912±0.015	3.202±0.102	4.543±0.155
	dAD(NN)	**0.913±0.015**	3.243±0.097	4.548±0.147
	RCP_0.05	0.881±0.006	1.598±0.023	2.690±0.125
	RCP_0.10	0.869±0.005	1.503±0.025	2.716±0.130
	RCP_0.30	0.863±0.012	**1.463±0.037**	2.762±0.127
	RCP_0.50	0.870±0.009	1.528±0.032	2.774±0.124
	RCP_0.75	0.879±0.006	1.605±0.019	2.772±0.122
0.2	CP	0.798±0.017	1.174±0.024	2.109±0.064
	CQR	0.791±0.013	1.216±0.045	2.101±0.061
	dAD(CV)	0.819±0.022	2.030±0.101	3.529±0.158
	dAD(NN)	**0.820±0.024**	2.062±0.101	3.535±0.158
	RCP_0.05	0.772±0.017	1.076±0.020	**2.045±0.069**
	RCP_0.10	0.761±0.016	1.050±0.023	2.064±0.070
	RCP_0.30	0.742±0.018	**1.004±0.024**	2.101±0.073
	RCP_0.50	0.749±0.019	1.023±0.027	2.112±0.072
	RCP_0.75	0.769±0.019	1.078±0.032	2.108±0.070

Bold values indicate the best performance. RCP_ρ denotes the proposed method with neighborhood proportion ρ, which determines the number of nearest neighbors used for local quantile estimation as a fraction of the calibration set.

4 Supplementary Figures

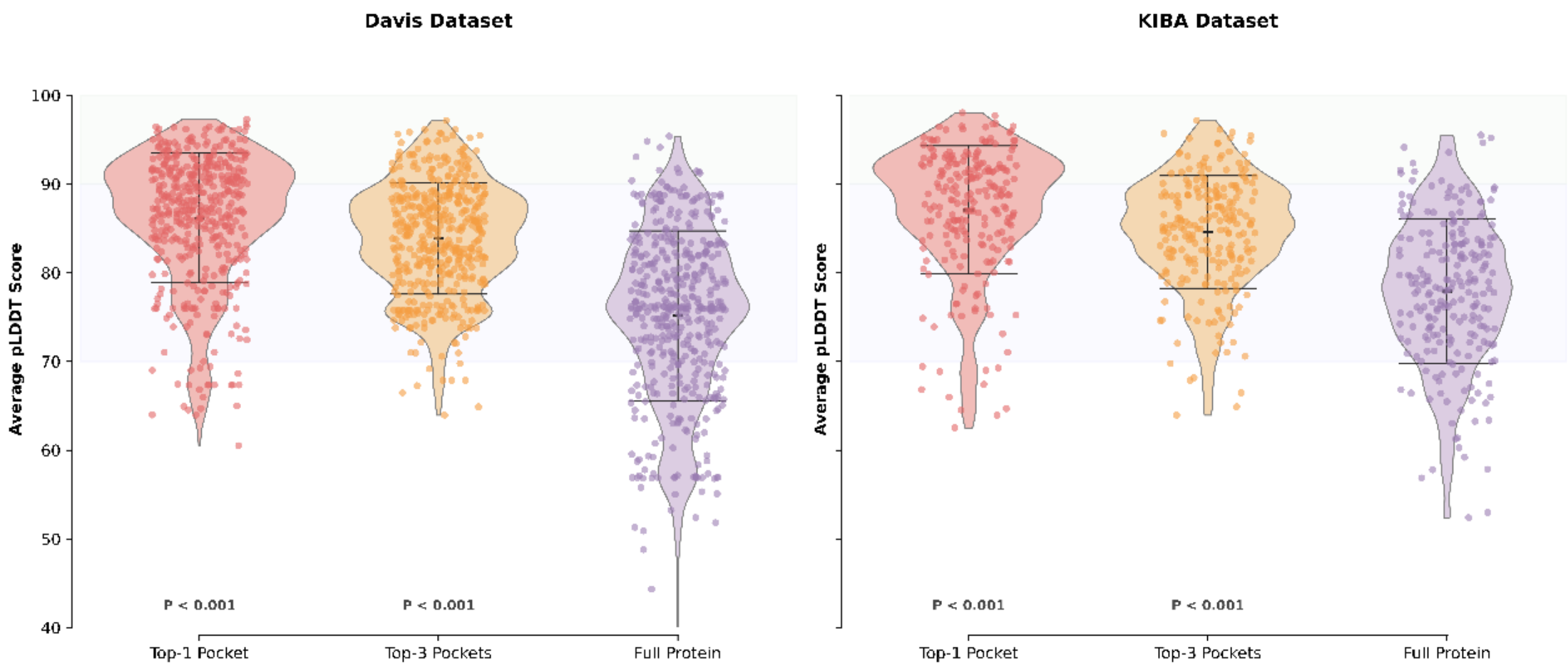

Figure S1. Statistical distribution of structural confidence scores across distinct protein regions.

The violin plots illustrate the distribution of pLDDT scores for full-length protein sequences compared to the top-ranked binding pockets (Top-1 and Top-3) identified by P2Rank in the Davis and KIBA datasets. Individual data points are superimposed to reveal the underlying density and variance. Statistical significance ($P < 0.001$) was assessed using Welch's t-test, which accounts for unequal variances. The results indicate that the predicted pocket regions exhibit significantly higher structural fidelity and local folding confidence compared to the full protein structure.

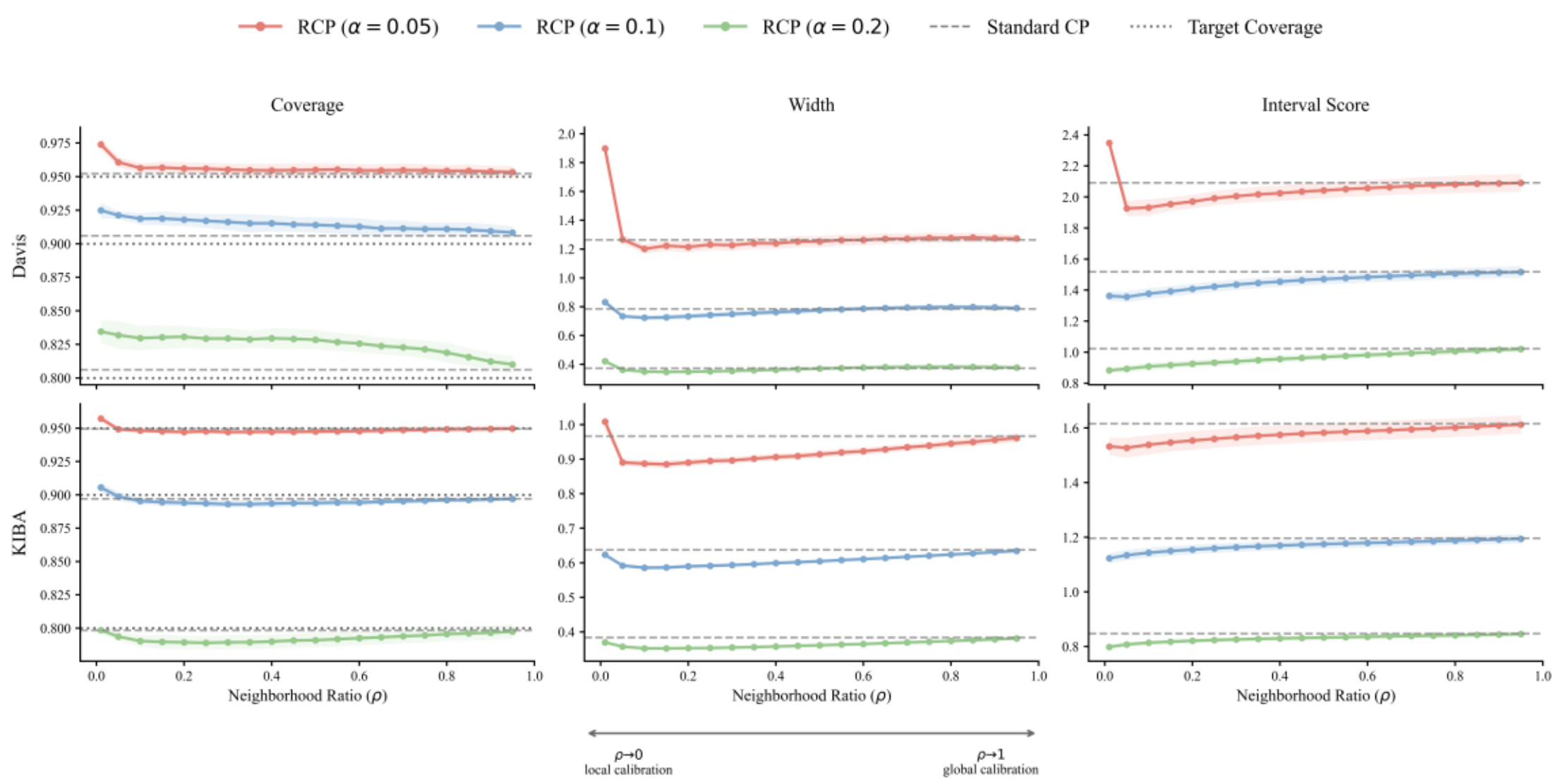

Figure S2. Sensitivity analysis of neighborhood ratio ρ on coverage, width, and interval score.

Sensitivity analysis of neighborhood ratio ρ showed that ρ mainly affects interval efficiency rather than coverage stability. Extremely small ρ values resulted in wider intervals due to limited calibration samples, while moderate ρ values (0.05–0.15) achieved lower Width and Interval Score, indicating more efficient uncertainty estimation. As ρ increased toward global calibration, interval efficiency gradually decreased due to increased neighborhood heterogeneity, whereas empirical coverage remained relatively stable across different settings.

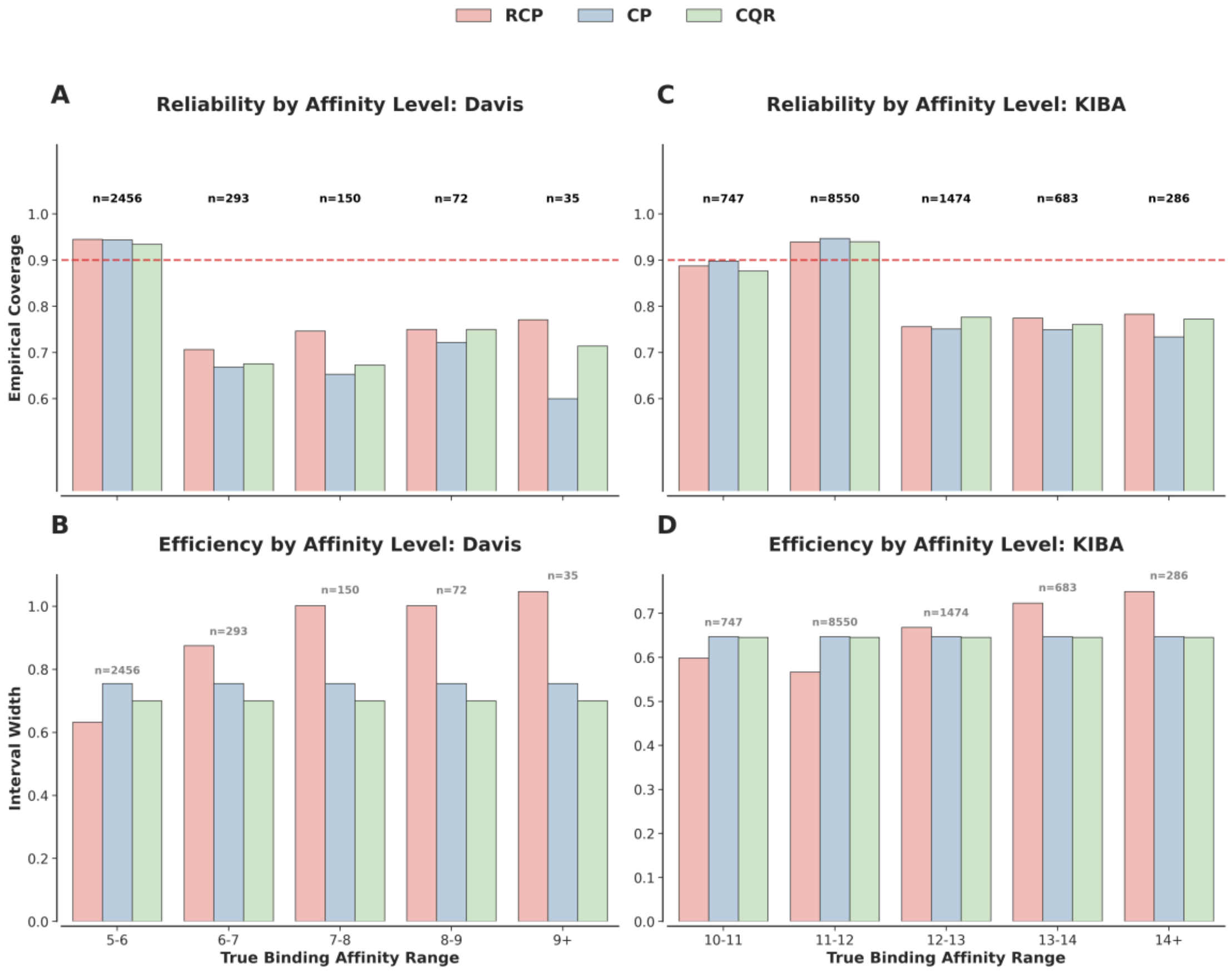

Figure S3. Reliability and efficiency analysis across different binding affinity levels. (A, C) Empirical coverage of RCP, CP, and CQR under different affinity ranges on Davis and KIBA datasets. (B, D) Corresponding interval widths.

Compared with global calibration methods, RCP-DTA achieves narrower intervals in regions with sufficient coverage while increasing interval widths in more challenging affinity regions where larger prediction uncertainty is observed. These results indicate that the retrieved local calibration samples provide informative uncertainty estimates for different regions of the affinity landscape.

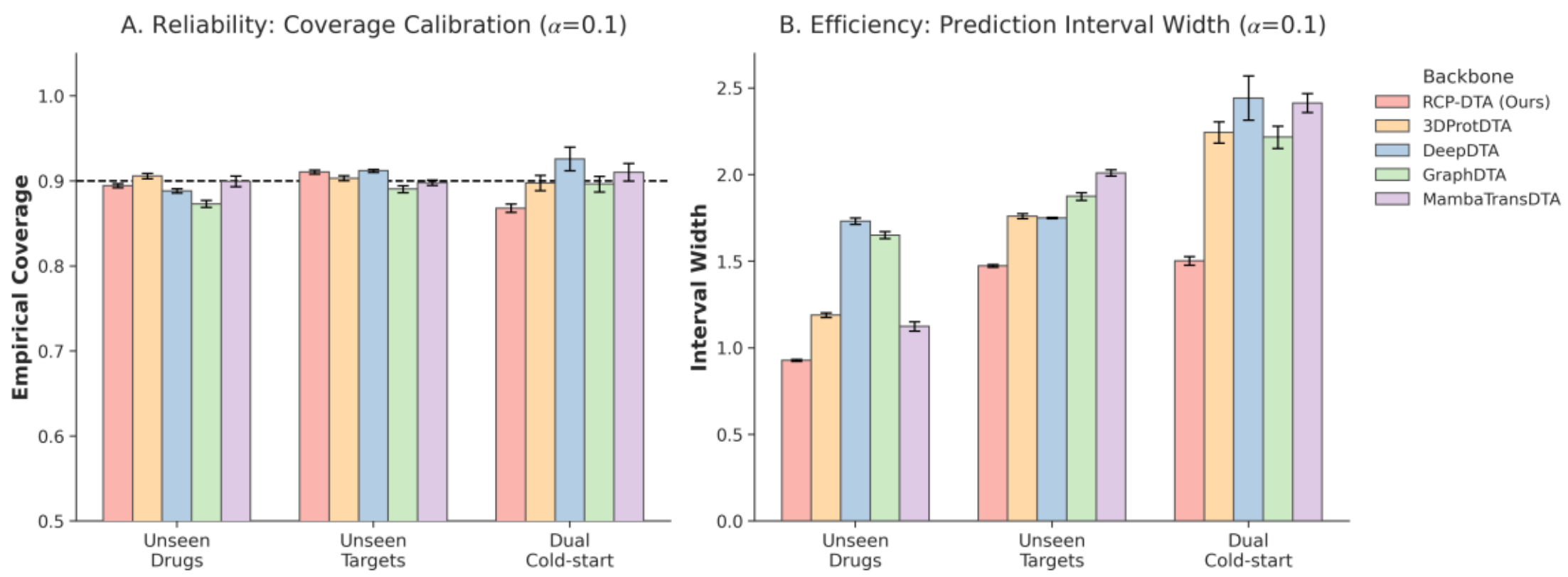

Figure S4. Reliability and efficiency evaluation of the RCP framework across diverse backbone architectures. (A) Empirical coverage calibration across OOD scenarios at α=0.10, where OOD evaluation is conducted on the KIBA dataset. (B) Average prediction interval width for the corresponding models.

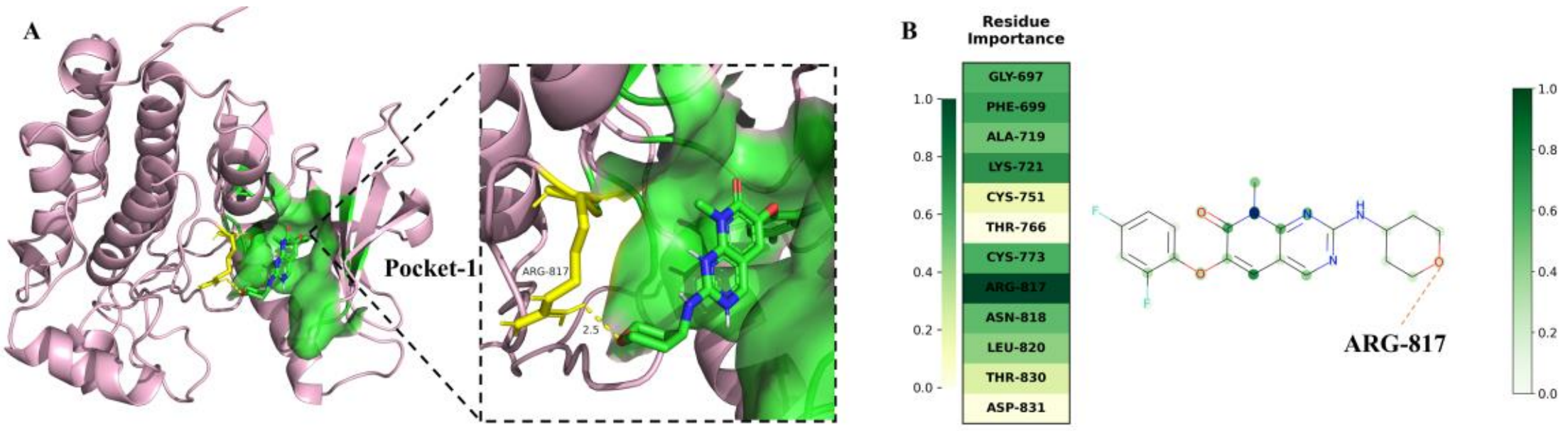

Figure S5. Structural validation and interpretability analysis of the EGFR-ligand interaction in Pocket-1. (A) Predicted binding conformation of the ligand within Pocket-1 of EGFR generated by AutoDock Vina. (B) Residue and atom-level importance analysis based on the Gradient×Input attribution method.

To further examine whether the proposed Virtual Pocket Feedback mechanism captures biologically meaningful binding regions, we conducted a structural case study using the representative low-uncertainty EGFR-ligand complex shown in Fig. 7B. The ligand (SMILES: Cn1c(=O)c(Oc2ccc(F)cc2F)cc2cnc(NC3CCOCC3)nc21) was docked into the EGFR crystal structure (PDB ID: 1M17) using AutoDock Vina (Eberhardt et al. 2021), followed by protein-ligand interaction analysis with PLIP (Schake et al. 2025). As illustrated in Supplementary Fig. S5 A-B, the predicted pocket substantially overlaps with the ligand-binding cavity identified by molecular docking. PLIP further detected four hydrophobic contacts (Phe699, Val702, Lys721, and Thr766), one hydrogen bond with Arg817, one π-π stacking interaction, and one π-cation interaction centered on Phe699. Encouragingly, the residues receiving the high Gradient×Input (Sundararajan et al. 2017) attribution scores, including Phe699, Lys721, and Arg817, coincide well with the interaction residues identified by PLIP. These observations provide independent structural evidence that the proposed Virtual Pocket Feedback mechanism guides the model toward biologically plausible binding regions.

References

Davis MI, Hunt JP, Herrgard S *et al.* Comprehensive analysis of kinase inhibitor selectivity. *Nat. Biotechnol.* 2011;**29**:1046-51.

Eberhardt J, Santos-Martins D, Tillack AF *et al.* AutoDock Vina 1.2.0: New docking methods, expanded force field, and python bindings. *J Chem Inf Model* 2021;**61**:3891-8.

Gilson MK, Liu T, Baitaluk M *et al.* BindingDB in 2015: A public database for medicinal chemistry, computational chemistry and systems pharmacology. *Nucleic Acids Res.* 2016;**44**:D1045-D53.

Krivák R and Hoksza D. P2Rank: machine learning based tool for rapid and accurate prediction of ligand binding sites from protein structure. *J. Cheminf.* 2018;**10**:39.

Liu L, Jiang H, He P *et al.* On the variance of the adaptive learning rate and beyond. arXiv, arXiv:1908.03265, 2019, preprint: not peer reviewed.

Loshchilov I and Hutter FJaL. SGDR: Stochastic gradient descent with warm restarts. arXiv, arXiv:1608.03983, 2016, preprint: not peer reviewed.

Metz JT, Johnson EF, Soni NB *et al.* Navigating the kinome. *Nat. Chem. Biol.* 2011;**7**:200-2.

Mirdita M, Schütze K, Moriwaki Y *et al.* ColabFold: making protein folding accessible to all. *Nat. Methods* 2022;**19**:679-82.

Paszke A, Gross S, Massa F *et al.* PyTorch: an imperative style, high-performance deep learning library. In: Adv Neural Inform Process Syst. NeurIPS, 2019, Article 721.

Schake P, Bolz SN, Linnemann K *et al.* PLIP 2025: introducing protein–protein interactions to the protein–ligand interaction profiler. *Nucleic Acids Res.* 2025;**53**:W463-W5.

Sun X, Liu X, Huang J *et al.* TSEDTA: a transformer-based neural network with SMILES transformer and ESM2 embeddings for drug-target binding affinity prediction. *Bioinformatics* 2026;**42**:btag298.

Sundararajan M, Taly A and Yan Q. Axiomatic attribution for deep networks. In: Proceedings of the 34th International Conference on Machine Learning. PMLR,

2017, 3319--28.

Tang J, Szwajda A, Shakyawar S *et al.* Making sense of large-scale kinase inhibitor bioactivity data sets: a comparative and integrative analysis. *J. Chem. Inf. Model.* 2014;**54**:735-43.

Varadi M, Bertoni D, Magana P *et al.* AlphaFold protein structure database in 2024: providing structure coverage for over 214 million protein sequences. *Nucleic Acids Res.* 2024;**52**:D368-D75.

Zhang MR, Lucas J, Hinton GE *et al.* Lookahead Optimizer: k steps forward, 1 step back. *Adv Neural Informat Process Syst* 2019;**32**.

Zhao L, Wang H and Shi S. PocketDTA: an advanced multimodal architecture for enhanced prediction of drug–target affinity from 3D structural data of target binding pockets. *Bioinformatics* 2024;**40**:btae594.
